# Genetic basis of nitrogen use efficiency and yield stability across environments in winter rapeseed

**DOI:** 10.1186/s12863-016-0432-z

**Published:** 2016-09-15

**Authors:** Anne-Sophie Bouchet, Anne Laperche, Christine Bissuel-Belaygue, Cécile Baron, Jérôme Morice, Mathieu Rousseau-Gueutin, Jean-Eric Dheu, Pierre George, Xavier Pinochet, Thomas Foubert, Olivier Maes, Damien Dugué, Florent Guinot, Nathalie Nesi

**Affiliations:** 1 INRA, UMR 1349 IGEPP, BP 35327, 35650 le Rheu, France; 2 AGROCAMPUS OUEST, UMR 1349 IGEPP, BP 35327, 35650 le Rheu, France; 3 Limagrain Europe, Ferme de l’Etang, 77390 Verneuil-l’Etang, France; 4 Biogemma, Chemin de Panedautes, 31700 Mondonville, France; 5 Terres Inovia, Avenue Lucien Brétignières, 78850 Thiverval Grignon, France; 6 Euralis, Chemin de Panedautes, 31700 Mondonville, France; 7 Maisadour Semences, Route de Saint Sever, BP27, 40001 Mont de Marsan Cedex, France; 8 RAGT R2n, Rue Emile Singla, BP 3331, 12033 Rodez, France; 9 Syngenta, Chemin de l’Hobit, 31790 Saint-Sauveur, France

**Keywords:** *Brassica napus* L, Nitrogen stress, Genotype × nitrogen interactions, Ecovalence, Quantitative trait loci

## Abstract

**Background:**

Nitrogen use efficiency is an important breeding trait that can be modified to improve the sustainability of many crop species used in agriculture. Rapeseed is a major oil crop with low nitrogen use efficiency, making its production highly dependent on nitrogen input. This complex trait is suspected to be sensitive to genotype × environment interactions, especially genotype × nitrogen interactions. Therefore, phenotyping diverse rapeseed populations under a dense network of trials is a powerful approach to study nitrogen use efficiency in this crop. The present study aimed to determine the quantitative trait loci (QTL) associated with yield in winter oilseed rape and to assess the stability of these regions under contrasting nitrogen conditions for the purpose of increasing nitrogen use efficiency.

**Results:**

Genome-wide association studies and linkage analyses were performed on two diversity sets and two doubled-haploid populations. These populations were densely genotyped, and yield-related traits were scored in a multi-environment design including seven French locations, six growing seasons (2009 to 2014) and two nitrogen nutrition levels (optimal versus limited). Very few genotype × nitrogen interactions were detected, and a large proportion of the QTL were stable across nitrogen nutrition conditions. In contrast, strong genotype × trial interactions in which most of the QTL were specific to a single trial were found. To obtain further insight into the QTL × environment interactions, genetic analyses of ecovalence were performed to identify the genomic regions contributing to the genotype × nitrogen and genotype × trial interactions. Fifty-one critical genomic regions contributing to the additive genetic control of yield-associated traits were identified, and the structural organization of these regions in the genome was investigated.

**Conclusions:**

Our results demonstrated that the effect of the trial was greater than the effect of nitrogen nutrition levels on seed yield-related traits under our experimental conditions. Nevertheless, critical genomic regions associated with yield that were stable across environments were identified in rapeseed.

**Electronic supplementary material:**

The online version of this article (doi:10.1186/s12863-016-0432-z) contains supplementary material, which is available to authorized users.

## Background

The worldwide demand for vegetable oils and proteins has significantly increased in recent decades due to population growth and increased standards of living. Therefore, high seed yield and quality are major goals in crop production, while at the same time, there is a need to stabilize seed production under fluctuating environments and to reduce the environmental impacts of agriculture by reducing the inputs. Rapeseed (*Brassica napus* L.) is a major oleaginous crop that is cultivated worldwide. It is grown for its oil-rich seeds (~40–45 % of the seed dry matter), which are used for food and industrial purposes, as well as for its seed cake containing ~30–35 % protein, which is used to feed livestock. Compared to other crops, rapeseed is highly demanding in terms of input, with particularly high requirements for mineral nitrogen (N) (~150–250 kg N/ha depending on the pedo-climatic growth conditions) for a seed yield of ~3.0–3.5 t/ha in Western Europe [1]. N fertilization is a key factor in the economic balance of rapeseed production, as N fertilizer is the main expense for farmers. In addition, there is serious concern regarding N loss in the field, which can lead to soil and water pollution through nitrate leaching and to air pollution through greenhouse gas emissions [2]. Reducing N input is therefore a current challenge for sustainable rapeseed production, which implies the maintenance of competitive yields at reduced N fertilization levels. This goal may be achieved by improving the nitrogen use efficiency (NUE), which can be defined as the process of converting N into seed yield [3].

Rapeseed is often described as a low-NUE crop, with values ranging from 15 to 20 kg seeds/kg of available N; the NUE of rapeseed is approximately half that of cereals (~35–40 kg seeds/kg N). The high oil accumulation in the seeds is an energy-consuming process requiring high amounts of carbon per unit of dry matter. This partly explains rapeseed relative low NUE [1]. In addition, the significant amounts of plant N that are lost through leaf fall during the crop cycle (approximately 45 kg N/ha) may also explain the low NUE of rapeseed [4].

From an agronomic point of view, the improvement of the NUE can be assessed by the increase of seed yield per unit of N fertilizer [5, 6]. Hence, a prerequisite for increasing NUE is gaining further insight into the genetic control of yield and yield components under contrasting N fertilization conditions. Additionally, the seed N content and the N harvest index are common proxies used to assess the efficiency of N remobilization from the vegetative to the reproductive organs and, more generally, to evaluate the NUE. However, a trade-off exists between the N and oil contents in seed, and this relationship must be uncoupled to increase the NUE while maintaining a high oil content. This uncoupling is one of the main goals of rapeseed breeding.

Yield is a particularly complex trait in rapeseed due to the plant’s capacity to grow and branch after flowering, which leads to compensations between the different yield components (seed number/m^2^, seed weight, etc.). Several quantitative trait loci (QTL) have already been identified as contributors to seed quality- and seed yield-associated traits in rapeseed [7–13]. However, only a few studies have reported on the genotypic yield stability under abiotic or nutritional constraints [14–16], particularly under sub-optimal N fertilization conditions [17–20]. This lack of evidence suggests that there is room to improve the understanding of the genetic control of NUE and yield stability across N nutrient conditions in rapeseed.

Gaining insight into this genetic control requires a better understanding of the genotypic responses to various N stress conditions. These responses are quantified by the genotype × N (G × N) interaction, which deviates from the expected trait level of one genotype under a particular N nutrient condition. The presence of G × N interactions may reflect specific genetic control depending on the N nutrient conditions. However, other biotic and abiotic stresses independent of crop N nutrient levels may occur throughout the crop cycle but are partially manageable with appropriate crop management. The combination of interactions of genotypes with all the stresses and/or constraints that are encountered throughout the crop cycle defines the genotype × environment (G × E) interactions.

Understanding the determinants of the G × E interactions for seed yield-related traits is a key consideration for breeding, and this issue has been extensively studied in crops [21]. Several parameters have been proposed to characterize the G × E interactions and to estimate phenotypic stability in multi-trial analyses; these proposals have been reviewed by Becker et al. [22]. Among them, non-parametric methods rely on genotype ranking between different environments [23]. Additional methods are based on the regression of each genotypic value according to either the means of the environments [24] or the environmental effects [25, 26], with the regression coefficients and the coefficients of determination used as indicators of genotypic stability. Finally, the calculation of ecovalence also provides clues to determine the contribution of each genotype to a G × E interaction [27]. All of these methods have been used to investigate G × E interactions and are likely to be transferable to the study of the G × N interactions. Nevertheless, the genetic determinants of these traits have hardly been studied to date [28].

The aims of the present study were to obtain a comprehensive overview of the genetic control of yield in winter oilseed rape and to assess the impact of N nutrition conditions on yield stability. To achieve these goals, a large variety of rapeseed genotypes were phenotyped in a wide network of trials under optimal versus limited N fertilization conditions calibrated to generate N stress and G × N interactions. We first studied the partition of the genotypic main effects: the G × N and G × trial interactions. We then combined genome-wide association studies (GWAS) and linkage analyses to investigate the genetic architecture of seed yield-related traits and the stability of these traits across environments by calculating ecovalence values. Finally, we assessed the genomic organization of the critical QTL within the *B. napus* genome.

## Methods

### Plant material and genotyping data

#### Populations for GWAS

A population of 92 WOSR accessions (hereafter referred to as the WOSR-92 population) was used for GWAS (Additional file 1: Table S1). The accessions originated from Western Europe, with 50 genotypes of the double-low type (‘00’, low in erucic acid and glucosinolates), 17 of the ‘0+’ type, 1 of the ‘+0’ type and 24 of the ‘++’ type. A subset of 69 individuals (WOSR-69) with homogeneous flowering precocities between accessions and a limited flowering period was chosen within the WOSR-92 set and considered for GWAS (Additional file 1: Table S1). All of the accessions were genotyped using the *Brassica* 60 K Infinium® SNP array (Illumina, Inc. San Diego, CA) [29], and the data were visualized using Genome Studio software (Illumina). Approximately 30 K SNPs were validated and scored in each of the WOSR populations using thresholds of 5 % for the minor allele frequency (MAF) and 10 % for the frequency of missing values (Additional file 2: Table S2). Up to 83 % of the SNPs were physically anchored to the *B. napus* genome [30], and most markers had a genetic position on the WOSR map that was obtained via successive projections of the individual maps of the Aviso × Montego, Tenor × Express, Darmor-*bzh* × Bristol, Aviso × Aburamasari and Darmor-*bzh* × Yudal crosses, all of which were genotyped using the *Brassica* 60 K SNP array (C. Falentin and G. Deniot, unpublished results). A pairwise estimate of linkage disequilibrium (LD, r^2^) was performed using PLINK 1.9 software [31, 32]. LD decay was evaluated using a non-linear regression of the expected r^2^ as described by Sved et al. [33] using the equation E[r^2^] = 1/(1 + 4 × Ne × c), where *c* is the recombination rate in morgans and *Ne* is the effective population size. E[r^2^] was plotted against the genetic distance between SNPs (in centimorgans (cM) or in base pairs (bp)) to estimate the extent of LD with the r^2^ set to 0.2. The LD decay of each WOSR population and of each linkage group is given in Additional file 2: Table S2. The genetic relatedness between individuals was assessed by computing an identity-by-state kinship matrix (K matrix) using the GEMMA package [34] with a set of 56 SSR markers spread uniformly across the genome [35, 36].

#### Populations for linkage analyses

Two populations of doubled haploid (DH) lines were derived from four WOSR lines with contrasting responses to different N fertilization conditions (unpublished data): Aviso × Montego (AM-DH, 112 individuals) and Tenor × Express (DK-DH, 75 individuals). The AM-DH population was described previously [19]. Both populations were genotyped with the *Brassica* 60 K SNP array using the same thresholds for SNP calling and validation as described for the WOSR populations. The AM-DH and DK-DH genetic maps contained 968 and 800 unique loci, covering a total length of 1,870 and 1,938 cM at a density of one locus per 1.93 and 2.42 cM, respectively.

##### Field trials

A summary of the different experimental conditions is shown in Table 1 and Additional file 3: Table S3. The trials (hereafter defined as combinations of location × year) were conducted in France across a set of locations representing a wide variety of pedo-climatic conditions. The WOSR-92 population was evaluated at Le Rheu (48.82163 N, 1.48926E) during the 2008–2009 (LR09) and 2009–2010 (LR10) crop seasons. The WOSR-69 population was evaluated in 2013–2014 at five sites: Châteauroux (Ch14, 46.914158 N, 1.756584E), Dijon (Dij14, 47.230468 N, 5.10036E), Prémesques (Pre14, 50.380000 N, 2.570000E), Selommes (Sel14, 47.44324 N, 1.14943E) and Verpillères (Ver14, 49.68028 N, 2.81528E). The AM-DH population was evaluated at Le Rheu in 2010–2011 (LR11), 2011–2012 (LR12) and 2012–2013 (LR13) as described previously [19]. The AM-DH population was also evaluated at Mondonville in 2010–2011 (Md11, 43.670000 N, 1.280000E), and a subset of 75 individuals was trialed in Dijon in 2012–2013 (Dij13, 47.234781 N, 5.104821E). The DK-DH population was evaluated at Le Rheu and Mondonville during the 2010–2011 crop season (LR11 and Md11, respectively).

**Table 1:** Experimental trials, crop management strategies and nitrogen nutrition indexes at the bolting stage (BBCH 50)

Population	Location	Year	Trial acronym	Number of individuals	∆N fertilization (a) (kg N/ha)	Mineral N soil content under plants at the end of winter (kg N/ha)	NNI (b)	N stress qualification
WOSR-92	Le Rheu	2008-2009	LR09	92	70	17	0.96 *(0.03) -* 1.31 *(0.13)*	No N stress
		2009-2010	LR10	92	70	17.1	0.97 *(NA) -* 1.17 *(0.001)*	No N stress
WOSR-69	Châteauroux	2013-2014	Ch14	69	100	-	0.81 *(0.19) -* 1.08 *(0.01)*	Moderate
	Dijon	2013-2014	Dij14	69	80	30.3	0.81 *(0.02) -* 0.93 *(0.01)*	Moderate
	Prémesques	2013-2014	Pre14	69	90	15.4	0.67 *(0.03) -* 1.02 *(0.01)*	Intense
	Selommes	2013-2014	Sel14	69	80	-	-	
	Verpillères	2013-2014	Ver14	69	60	-	0.87 *(0.02) -* 1.14 *(0.05)*	Moderate
AM-DH	Le Rheu	2010-2011	LR11	112	90	11	0.97 *(0.18) -* 0.93 *(0.07)*	No N stress
		2011-2012	LR12	112	80	30.3	0.96 *(0.19) -* 1.08 *(0.27)*	No N stress
		2012-2013	LR13	112	80	52.5	0.81 *(0.08) -* 1.12 *(0.05)*	Moderate
	Mondonville	2010-2011	Md11	112	90	14.1	0.84 *(0.08) -* 1.08 *(0.09)*	Moderate
	Dijon	2012-2013	Dij13	75	60	40.6	0.98 *(0.06) -* 1.04 *(0.04)*	No N stress
DK-DH	Le Rheu	2010-2011	LR11	75	80	11	0.97 *(0.18) -* 0.93 *(0.07)*	No N stress
	Mondonville	2010-2011	Md11	75	90	14.1	0.84 *(0.08) -* 1.08 *(0.09)*	Moderate

(a)∆N fertilization corresponds to the difference between the N fertilization under the high (N2) and low (N1) conditions

(b)The nitrogen nutrition index measured at the bolting stage (BBCH 50) under low (N1, left) or high (N2, right) N nutrition conditions. The standard errors are indicated in brackets

'-': not available

Plants were grown under two N nutrition conditions (N1: low; N2: optimal) as described in detail below. To limit the amount of mineral N in the soil in the experimental plots, no organic matter was spread on the fields for three years before the trials, and the previous crops were grown under a low-N-input management system. All of the trials were designed as split plots with N as the main plot and genotypes as the sub-plots, except for the Md11 trials, which were designed as alpha plans with N nutrient conditions as the main plots and genotypes as the sub-plots (Additional file 3: Table S3). Seeds were sown in plots of 10 to 18 m^2^ at a density of 35 plants/m^2^. In each trial, control plots planted with the Aviso *cv*. were included in the design to assess the N status of the plants throughout the crop cycle using N nutrition index (NNI) measurements according to Colnenne et al. [38] (see below). The mineral soil content was measured as described previously just before sowing, at the end of winter and just after harvest [19].

N fertilization was calculated using the balance sheet method, which is commonly used in France for the main arable crops [39, 40]. The difference in fertilizer amounts between the two N treatments varied between 60 and 100 kg N/ha, depending on the trial (Table 1, Additional file 3: Table S3). All of the N applications were made using a liquid fertilizer solution containing 39 % N (50 % urea, 25 % nitrate and 25 % ammonium) on two dates (the beginning of stem elongation and during spring elongation), except for Dij13 and Sel14, for which an additional application was made at the very beginning of flowering (Additional file 3: Table S3).

For each trial, the NNI was measured at three time points, including the end of the autumnal period (BBCH 19: date 1), the end of the winter period (BBCH 30: date 2) and during the course of spring elongation (BBCH 50: date 3) (Additional file 3: Table S3). On dates 1 and 2, no N fertilizer was applied so that all of the plants were at the same N nutrition level. Only the NNI values at BBCH 50 are presented in this study. The plants were considered stressed if the NNI values were below 0.90, and the intensity of the stress increased as the NNI value decreased. Intense stress conditions were defined as NNI values below 0.75. The N stresses that were applied to the crops were moderate for five of the trials, including LR13, Md11, Ch14, Dij14 and Ver14; in these trials, the NNI values for the low-N conditions ranged from 0.81 to 0.87. The N stress was intense in the Pre14 trial (NNI__N1_ = 0.67), whereas no N stress was detected in the other trials (NNI__N1_ > 0.96) (Table 1). However, despite the absence of N stress, differences in NNI values were observed between the two N treatments for the LR09 and LR10 trials (ΔNNI of 0.35 and 0.2, respectively), reflecting differences in plant N nutrition status between N fertilization conditions.

### Phenotypic data acquisition and analysis

The measured traits were previously described in detail [19] and were as follows: days to flowering (DTF in days, measured at BBCH 61 [37]), seed yield (SY in t/ha), thousand-seed weight (TSW in g), seed number/m^2^ (SN = (SY × 100,000)/TSW), seed oil content (O in % of seed dry matter), seed protein content (Pr in % of seed dry matter) and seed oil content/seed protein content ratio (O/Pr). All of the statistical analyses were carried out with R software version 3.2.4 [41].

#### Characterization of the trials

To characterize the different environments (hereafter defined as combinations of trial × N treatment), a principal component analysis (PCA) was performed on the phenotypic means of each genotype for the AM-DH and WOSR-69 populations. The environments were then grouped via hierarchical clustering based on the coordinates of each genotype on the first five principal components (FactoMineR package; [42]). For the AM-DH population, data from Dij13 were not considered for the clustering analysis because the DTF, SN and TSW traits were not recorded in this trial. The clustering of environments was not performed for the DK-DH population because it was only tested in two trials (LR11 and Md11) that were already addressed in the AM-DH population. Concerning the WOSR-69 population, the DTF trait was not considered because it was not recorded in Ver14, and the data from Dij14 were discarded from the analysis because DTF, SY and SN were not recorded in this trial. The phenotypic and genetic correlations (r_g_) between the traits averaged over the trials for each population and each N fertilization condition were also calculated.

#### Mixed linear models

Different mixed models were analyzed using the lme4 [43] and lmerTest [44] packages, and the results are presented below.

First, a mixed linear model was applied to each trait (P) using the REML method, with all of the trials and N fertilization conditions confounded. This multi-environment model (1) was fitted for the two DH populations as well as for the WOSR-69 population tested in seven trials (LR09, LR10, Ch14, Dij14, Pre14, Sel14, and Ver14):

1 $$ {\boldsymbol{P}}_{\boldsymbol{i}\boldsymbol{jklm}} = \boldsymbol{\mu} +\underline {{\boldsymbol{G}}_{\boldsymbol{i}}} + {\boldsymbol{N}}_{\boldsymbol{j}} + {\underline {\boldsymbol{T}}}_{\boldsymbol{l}}+\underline {{\boldsymbol{T}}_{\boldsymbol{l}}\left({\boldsymbol{R}}_{\boldsymbol{k}}\right)} + \underline {{\boldsymbol{G}}_{\boldsymbol{i}}\times {\boldsymbol{N}}_{\boldsymbol{j}}}+{\underline {{\boldsymbol{G}}_{\boldsymbol{i}} \times \boldsymbol{T}}}_{\boldsymbol{l}} + {\underline {{\boldsymbol{G}}_{\boldsymbol{i}}\times {\boldsymbol{N}}_{\boldsymbol{j}}\times \boldsymbol{T}}}_{\boldsymbol{l}} + \underline {{\boldsymbol{e}}_{\boldsymbol{i}\boldsymbol{jklm}}} $$

where *P*_*ijklm*_is the phenotypic value, *μ* is the population mean, *G*_*i*_ is the genotype *i*, *N*_*j*_ is the N nutrition condition *j*, *R*_*k*_ is the replicate *k*, *T*_*l*_ is the trial *l*, and *e*_*ijklm*_ is the residual. The underlined terms were considered as random. Based on model (1), broad sense heritability (h^2^) was then calculated as follows:

2 $$ {\boldsymbol{h}}^2=\frac{{{\boldsymbol{\sigma}}^2}_{\boldsymbol{G}}}{{{\boldsymbol{\sigma}}^2}_{\boldsymbol{G}} + \frac{{{\boldsymbol{\sigma}}^2}_{\boldsymbol{G}\times \boldsymbol{N}}}{\boldsymbol{n}} + \frac{{{\boldsymbol{\sigma}}^2}_{\boldsymbol{G}\times \boldsymbol{T}}}{\boldsymbol{t}} + \frac{{{\boldsymbol{\sigma}}^2}_{\boldsymbol{e}}}{\boldsymbol{t}\times \boldsymbol{n}\times \boldsymbol{r}}} $$

where *σ*^*2*^_*G*_is the genetic variance, *σ*^*2*^_*G×N*_ is the G × N variance, *σ*^*2*^_*e*_ is the residual variance, *σ*^*2*^_*G×T*_ is the G × T variance, *n* is the number of N fertilization conditions, *t* is the number of trials, and *r* is the number of replicates per genotype, per N fertilization condition, and per trial.

A second mixed linear model was applied to each trait (P) in each trial, with all N conditions confounded. Model (3) was adjusted for trials with a split plot design:

3 $$ {\boldsymbol{P}}_{\boldsymbol{i}\boldsymbol{jkl}} = \boldsymbol{\mu} +\underline {{\boldsymbol{G}}_{\boldsymbol{i}}} + {\boldsymbol{N}}_{\boldsymbol{j}} + \underline {{\boldsymbol{R}}_{\boldsymbol{k}}} + \underline {{\boldsymbol{G}}_{\boldsymbol{i}}\times {\boldsymbol{N}}_{\boldsymbol{j}}} + \underline {{\boldsymbol{e}}_{\boldsymbol{i}\boldsymbol{jkl}}} $$

Model (4) was fitted for the trials with an alpha plan design (AM-DH and DK-DH in Md11 trials):

4 $$ {\boldsymbol{P}}_{\boldsymbol{i}\boldsymbol{jklm}} = \boldsymbol{\mu} +\underline {{\boldsymbol{G}}_{\boldsymbol{i}}} + {\boldsymbol{N}}_{\boldsymbol{j}} + \underline {{\boldsymbol{R}}_{\boldsymbol{k}}}+\underline {{\boldsymbol{R}}_{\boldsymbol{k}}\left({\boldsymbol{B}}_{\boldsymbol{l}}\right)}+\underline {{\boldsymbol{G}}_{\boldsymbol{i}}\times {\boldsymbol{N}}_{\boldsymbol{j}}} + \underline {{\boldsymbol{e}}_{\boldsymbol{i}\boldsymbol{jklm}}} $$

where *P*_*ijkl*_ and *P*_*ijklm*_ are the phenotypic values, *μ* is the population mean, *G*_*i*_ is the genotype *i*, *N*_*j*_ is the N nutrition condition *j*, *R*_*k*_ is the replicate *k*, *B*_*l*_ is the block *l*, and *e*_*ijkl*_ and *e*_*ijklm*_ are residuals. All of the effects were considered random except for the N nutrition effect.

The corresponding heritabilities were assessed as follows:

5 $$ {\boldsymbol{h}}^2=\frac{{{\boldsymbol{\sigma}}^2}_{\boldsymbol{G}}}{{{\boldsymbol{\sigma}}^2}_{\boldsymbol{G}} + \frac{{{\boldsymbol{\sigma}}^2}_{\boldsymbol{G}\times \boldsymbol{N}}}{\boldsymbol{n}} + \frac{{{\boldsymbol{\sigma}}^2}_{\boldsymbol{e}}}{\boldsymbol{n}\times \boldsymbol{r}}} $$

Finally, a random linear model was applied to each trait P for each trial and N fertilization condition. This single-environment model (6) was fitted for each population (WOSR-92, WOSR-69, AM-DH and DK-DH):

6 $$ {\boldsymbol{P}}_{\boldsymbol{i}\boldsymbol{jk}} = \boldsymbol{\mu} +\underline {{\boldsymbol{G}}_{\boldsymbol{i}}} + \underline {{\boldsymbol{R}}_{\boldsymbol{j}}} + \underline {{\boldsymbol{e}}_{\boldsymbol{i}\boldsymbol{jk}}} $$

where *P*_*ijk*_ is the phenotypic value, *μ* is the population mean, *G*_*i*_ is the genotype *i*, *R*_*j*_ is the replicate *j*, and *e*_*ijk*_ is the residual. All of the terms were considered as random. Additionally, h^2^ was estimated for each N fertilization condition and each trial using the following formula:

7 $$ {\boldsymbol{h}}^2=\frac{{{\boldsymbol{\sigma}}^2}_{\boldsymbol{G}}}{{{\boldsymbol{\sigma}}^2}_{\boldsymbol{G}} + \frac{{{\boldsymbol{\sigma}}^2}_{\boldsymbol{e}}}{\boldsymbol{r}}} $$

#### Stability of the genotypes across environments

The stability of the genotypes from a given population across N fertilization conditions or trials was estimated by calculating the corresponding ecovalence values as described by Wricke (1962) [27]:

8 $$ {\boldsymbol{W}}_{\boldsymbol{i}} = {\displaystyle {\sum}_{\boldsymbol{i}}}{\left({\boldsymbol{Y}}_{\boldsymbol{i}\boldsymbol{j}}-{\boldsymbol{Y}}_{\boldsymbol{i}.}-{\boldsymbol{Y}}_{.\boldsymbol{j}}+{\boldsymbol{Y}}_{..}\ \right)}^2 $$

where *Y*_*ij*_ is the phenotypic value of genotype *i* under treatment *j* (N nutrition condition or trial), *Y*_*i.*_ is the mean phenotypic value of genotype *i* over all of the considered treatments (all N nutrition conditions or trials), *Y*_*.j*_ is the mean phenotypic value of treatment *j* (N nutrition condition or trial), and *Y*_*..*_ is the general mean. The ecovalence calculated over the N fertilization conditions was called the G × N model, and the ecovalence calculated over the trials was called the G × T model.

### Genetic analyses

For GWAS, a compressed mixed linear model [45] implemented in the GAPIT R package [46] was used. For each genotype of the WOSR populations, four datasets were considered for the GWAS of a given trait: 1) the adjusted means extracted from the single-environment model (6), 2) the genotypic estimates across trials extracted from the multi-environment model (1), 3) the ecovalence values over the N fertilization conditions extracted from the G × N model (8), and 4) the ecovalence values over the trials extracted from the G × T model (8). A mixed linear model (MLM) in which the K matrix was declared to be random was applied to each of the analyses, and fixed marker effects were included one by one. To correct for multiple analyses, the false discovery rate (FDR) was calculated for each test as previously described [47], and SNPs with a FDR of less than 0.15 were considered significantly associated with a given trait. To define trait-associated genomic regions (GWAS-QTL), confidence intervals were calculated as described by Cormier et al. [48]. Briefly, the trait-associated SNPs were clustered according to their genetic relatedness, and the boundaries of each cluster were extended via the addition of the local LD decay value, calculated with all of the markers covering 5 % of the linkage group length from the middle of the cluster. In addition, the SNP with the lowest FDR within each cluster was considered the most probable position of the GWAS-QTL.

For linkage analyses, a multiple QTL mapping (MQM) model was tested using the R/qtl package [49]. For each genotype of the DH populations, the same four datasets as those described above for GWAS were considered. The QTL mapping models were previously described in detail [19], and a p-value of 0.05 was considered the threshold for significance. The trait-associated genomic regions arising from the linkage analyses were referred to as LA-QTL. GWAS-QTL and LA-QTL were finally projected onto the WOSR map using BioMercator software [50].

### Genomic analyses of targeted regions

Trait-associated QTL were analyzed in terms of structural organization within the *B. napus* genome. For this purpose, we focused on the QTL detected with the multi-environment model (1), which were associated with yield components (DTF, SY, SN, and TSW). The homoeologous relationships between genes from the A and C sub-genomes were extracted from the structural annotation of the Darmor-*bzh* genome sequence published by Chalhoub et al. [30] and aligned with the physical positions of the QTL found in the present study to find consistent matches. The results were represented graphically using CIRCOS [51].

## Results

### Yield-related traits were highly heritable

Broad sense heritability values calculated with the multi-environment model (h^2^, model (2)) were always greater than 0.84 for all traits in all populations, with the exception of the DK-DH population, in which h^2^ decreased to 0.63 (Table 2). Similar assessments were observed when considering the trait heritability values within each population and trial combination (h^2^, model (5)). In this case, h^2^ was high and was always greater than 0.8, except for the Md11 trials (Additional file 4: Table S4). When the traits were considered in each population per trial × N combination, h^2^ (model (7)) was high for all of the traits, with generally higher h^2^ for the N2 condition than for the N1 condition (Additional file 4: Table S4).

**Table 2:** Results of the mixed linear model applied to each population for each trait considering all trials and N conditions as confounded (multi-environment model (1)), ${\boldsymbol{P}}_{\boldsymbol{i}\boldsymbol{jklm}} = \boldsymbol{\mu} +\underline {{\boldsymbol{G}}_{\boldsymbol{i}}} + {\boldsymbol{N}}_{\boldsymbol{j}} + \underline {{\boldsymbol{T}}_{\boldsymbol{l}}}+\underline {{\boldsymbol{T}}_{\boldsymbol{l}}\left({\boldsymbol{R}}_{\boldsymbol{k}}\right)} + \underline {{\boldsymbol{G}}_{\boldsymbol{i}}\times {\boldsymbol{N}}_{\boldsymbol{j}}}+\underline {{\boldsymbol{G}}_{\boldsymbol{i}} \times {\boldsymbol{T}}_{\boldsymbol{l}}} + {\underline {{\boldsymbol{G}}_{\boldsymbol{i}}\times {\boldsymbol{N}}_{\boldsymbol{j}}\times \boldsymbol{T}}}_{\boldsymbol{l}} + \underline {{\boldsymbol{e}}_{\boldsymbol{i}\boldsymbol{jklm}}}$

				T		N		G		G × N		G × T		G × N × T		h^2^ (c)
		Mean values ± standard error	Number of trials (a)	Var (b)	p_value_	Var	p_value_	Var	p_value_	Var	p_value_	Var	p_value_	Var	p_value_	
DTF	WOSR	96.32 ± 10.29	7	83.62	***	16.86	***	7.15	***	<10^−16^	NS	2.56	***	0.27	***	0.94
	AM-DH	85.25 ± 6.55	4	29.9	***	7.65	***	16.42	***	<10^−16^	NS	3.16	***	9.30.10^−2^	*	0.95
	DK-DH	83.87 ± 5.64	2	28.85	***	14.65	***	15.13	***	<10^−16^	NS	3.21	***	7.54.10^−2^	NS	0.90
SY	WOSR	2.75 ± 1.07	7	0.66	***	0.12	***	0.31	***	1.01.10^−3^	NS	0.11	***	1.79.10^−2^	**	0.92
	AM-DH	3.32 ± 0.66	5	2.41	***	5.05	***	0.5	***	<10^−16^	NS	0.23	***	0.44	***	0.85
	DK-DH	2.68 ± 0.63	2	1.85	***	9.32	***	0.62	***	<10^−16^	NS	0.51	***	0.25	***	0.63
TSW	WOSR	4.64 ± 0.54	7	4.45.10^−2^	***	9.40.10^−3^	***	0.17	***	2.56.10^−3^	**	2.56.10^−2^	***	6.47.10^−3^	***	0.97
	AM-DH	4.37 ± 0.70	5	0.75	***	0.19	**	4.52.10^−2^	***	<10^−16^	NS	1.69.10^−2^	***	3.85.10^−3^	***	0.92
	DK-DH	3.67 ± 0.58	2	0.51	***	0.25	NS	3.20.10^−2^	***	1.07.10^−3^	NS	2.01.10^−2^	***	7.10.10^−4^	NS	0.73
SN	WOSR	59,925 ± 24,740	7	3.36.10^8^	***	5.95.10^7^	***	1.66.10^8^	***	1.13.10^−6^	NS	5.74.10^7^	***	7.48.10^6^	*	0.92
	AM-DH	75,428 ± 18,007	5	2.35.10^8^	***	3.97.10^7^	***	2.80.10^7^	***	<10^−16^	NS	1.47.10^7^	***	3.39.10^7^	***	0.84
	DK-DH	73,614 ± 16,275	2	7.46.10^6^	**	1.03.10^7^	***	7.41.10^7^	***	2.58.10^6^	NS	3.65.10^7^	***	9.65.10^6^	NS	0.73
Pr	WOSR	18.61 ± 2.59	7	4.87	***	0.74	***	1.48	***	<10^−16^	NS	0.32	***	0.16	***	0.94
	AM-DH	19.93 ± 2.27	5	4.89	***	0.99	***	0.25	***	2.97.10^−14^	NS	4.53.10^−2^	*	0.16	***	0.90
	DK-DH	19.08 ± 3.04	2	-	-	-	-	-	-	-	-	-	-	-	-	-
O	WOSR	49.26 ± 2.43	7	1.83	***	0.31	***	2.17	***	8.49.10^−3^	NS	0.58	***	0.12	***	0.94
	AM-DH	47.94 ± 3.36	5	14.11	***	2.87	***	0.6	***	<10^−16^	NS	0.19	***	0.18	***	0.91
	DK-DH	48.73 ± 4.09	2	-	-	-	-	-	-	-	-	-	-	-	-	-
O/Pr	WOSR	2.71 ± 0.46	7	0.11	***	1.75.10^−2^	***	5.81.10^−2^	***	<10^−16^	NS	1.72.10^−2^	***	7.32.10^−3^	***	0.95
	AM-DH	2.46 ± 0.45	5	0.22	***	4.49.10^−2^	***	8.66.10^−3^	***	<10^−16^	NS	3.44.10^−3^	***	2.68.10^−3^	***	0.87
	DK-DH	2.65 ± 0.60	2	-	-	-	-	-	-	-	-	-	-	-	-	-

The significance of the genotype (G), nitrogen level (N), trial (T) and their interactions (G × N, G × T, G × N × T) was assessed for each population

(***, *p*-value < 0.001; **, 0.01 < *p*-value < 0.001; *, 0.05 < *p*-value; NS, non-significant; '-', not available)

(a)Number of trials considered for evaluation of the different effects

(b)Var: Variance components

(c)Heritabilities (h^2^) were calculated according to model (2): ${\mathrm{h}}^2=\frac{{\sigma^2}_G}{{\sigma^2}_G+\frac{{\sigma^2}_G\kern0.1em \mathrm{x}\kern0.1em \mathrm{N}}{\mathrm{n}}+\frac{{\sigma^2}_G\kern0.1em \mathrm{x}\kern0.1em \mathrm{T}}{\mathrm{T}}+\frac{{\sigma^2}_{\mathrm{e}}}{\mathrm{t}\;\mathrm{x}\;\mathrm{n}\;\mathrm{x}\;\mathrm{r}}}$ for each population; all environments (N × T) were considered as confounded

In addition, several of the traits showed strong correlations. For instance, the seed number/m^2^ was positively correlated with the seed yield (0.62 < r_p_ < 0.93), with strong positive genetic correlations (0.85 < r_g_ < 1.26) for all of the populations and all of the trials studied (Additional file 5: Table S5). As already known from previous studies, oil and protein contents always displayed strong negative correlations (−0.81 < r_p_ < −0.34; −1.14 < r_g_ < −0.54 in our study).

### NUE and yield traits were strongly impacted by N, trial and G × trial interaction effects, whereas weak G × N interactions were observed

When considering the multi-environment model (1), significant genotype (G), trial (T) and G × T interaction effects were found for each trait in each of the populations (Table 2). A significant N effect was also detected for each trait × population combination, except for TSW in the DK-DH population. However, no significant G × N effect was detected regardless of the trait and population considered, except for TSW in the WOSR population. Finally, significant G × T × N effects were observed in almost all cases, except for DTF, TSW and seed number/m^2^ in the DK-DH population.

When considering models (3) and (4) for each population × trial combination, the G effects were always highly significant, and N had an effect on most of the traits (Additional file 4: Table S4). Moreover, some G × N interactions were detected with these models, although they were not highly significant for most of the traits (0.01 < *p*-value < 0.05). In addition, the G × N interactions were detected in the LR09 and Ch14 trials for the WOSR populations and in the Md11 trial for the DH populations. These results prompted us to obtain further insight into the genetic control of these traits for each population and each trial under N1 and N2 conditions.

### Genetic analyses based on single-environment models revealed a high number of stable QTL between N nutrition conditions that were mostly trial-specific

The architecture of the genetic control of the seed yield and the genomic stability across environments was first assessed by analyzing the QTL detected in the single-environment model (6). A total of 946 GWAS-QTL were detected in the WOSR populations (486 and 460 for WOSR-92 and WOSR-69, respectively; Additional file 6: Table S6), and 184 LA-QTL were detected in the DH populations (138 and 46 for AM-DH and DK-DH, respectively; Additional file 7: Table S7). Most of the QTL were specific to a population structure, with only 35 loci in common between the DH and WOSR populations. In particular, one region located in the A5 linkage group was detected in the AM-DH and WOSR populations under both N fertilization conditions in 13 different environments (data not shown). In addition, a striking result was the significant proportion of loci controlling flowering time in the WOSR-92 population (63 and 12 % of the GWAS-QTL were associated with DTF in LR09 and LR10, respectively), as well as in the two DH populations (34 and 43 % in AM-DH and DK-DH, respectively). In contrast, no DTF-associated QTL were detected in the WOSR-69 population due to the lower MAF in this smaller population at the loci identified in the WOSR-92 population (data not shown). The DTF-associated QTL were generally highly stable across environments (data not shown).

The stability of the QTL for yield-related traits between N treatments was consistent with the level of N stress observed in each trial (Table 3). Indeed, for most of the trials in which no N stress was noted (i.e., LR09, LR10, LR11 and LR12), a large proportion of the QTL (37 to 70 %) were independent of the N fertilization level. In contrast, when the N stress was moderate to intense, as for LR13, Md11, Ch14, Dij14 and Pre14, a lower proportion of N-stable QTL (22 to 48 %) was found than when no N stress was present. However, there was one exception: in Dij13, a no-stress trial, only 18 % of the QTL were common between the two N treatments. These results suggest that additional environmental stresses occurred during the trials and that these additional factors interacted with the N nutrition level. We also examined QTL consistency across trials and showed that more than 50 % of the QTL were specific to a single trial (data not shown).

**Table 3:** Number of significant QTL detected for each trial × N combination and the consistency of the QTL across N nutrient conditions for the WOSR (A) and DH (B) populations

A (WOSR populations)			SY	TSW	SN	Pr	O	O/Pr	Sum
LR09	N1^a^		18	0	0	47	39	40	144
	N2^b^		25	0	0	45	43	42	155
	Total^c^		43	0	0	92	82	82	299
	N1-specific^d^		8 *(0.19)*	0	0	16 *(0.17)*	13 *(0.16)*	16 *(0.20)*	53 *(0.18)*
	N2-specific^e^		13 *(0.30)*	0	0	14 *(0.15)*	17 *(0.21)*	18 *(0.22)*	62 *(0.21)*
	Consistent^f^		22 *(0.51)*	0	0	62 *(0.68)*	52 *(0.63)*	48 *(0.58)*	184 *(0.61)*
LR10	N1		0	0	0	0	7	9	16
	N2		7	0	8	2	13	8	38
	Total		7	0	8	2	20	17	54
	N1-specific		-	0	-	-	2 *(0.10)*	4 *(0.23)*	6 *(0.11)*
	N2-specific		7 *(1.00)*	0	8 *(1.00)*	2 *(1.00)*	8 *(0.40)*	3 *(0.18)*	28 *(0.52)*
	Consistent		0	0	-	-	10 *(0.50)*	10 *(0.59)*	20 *(0.37)*
Ch14	N1		0	0	0	17	23	18	58
	N2		28	14	1	4	11	5	63
	Total		28	14	1	21	34	23	121
	N1-specific		0	0	0	13 *(0.62)*	13 *(0.38)*	13 *(0.56)*	39 *(0.32)*
	N2-specific		28 *(1.00)*	14 *(1.00)*	1 *(1.00)*	0 *(0)*	1 *(0.03)*	0 *(0)*	44 *(0.36)*
	Consistent		0	0	0	8 *(0.38)*	20 *(0.59)*	10 *(0.44)*	38 *(0.32)*
Dij14	N1		-	-	-	46	52	37	135
	N2		-	-	-	7	14	7	28
	Total		-	-	-	53	66	44	163
	N1-specific		-	-	-	40 *(0.75)*	42 *(0.64)*	33 *(0.75)*	115 *(0.70)*
	N2-specific		-	-	-	1 *(0.02)*	4 *(0.06)*	3 *(0.07)*	8 *(0.05)*
	Consistent		-	-	-	12 *(0.23)*	20 *(0.30)*	8 *(0.18)*	40 *(0.25)*
Pre14	N1		2	0	0	3	3	3	11
	N2		0	41	0	3	4	3	51
	Total		2	41	0	6	7	6	62
	N1-specific		2 *(1.00)*	0	0	0	0	0	2 *(0.03)*
	N2-specific		0	41 *(1.00)*	0	0	1 *(0.14)*	0	42 *(0.68)*
	Consistent		0	0	0	6 *(1.00)*	6 *(0.86)*	6 *(1.00)*	18 *(0.29)*
Sel14	N1		8	0	1	0	0	0	8
	N2		46	0	0	0	0	0	47
	Total		54	0	1	0	0	0	55
	N1-specific		0	0	1 *(1.00)*	0	0	0	0
	N2-specific		38 *(0.70)*	0	0	0	0	0	39 *(0.71)*
	Consistent		16 *(0.30)*	0	0	0	0	0	16 *(0.29)*
Ver14	N1		5	0	1	6	0	2	13
	N2		45	0	0	0	0	0	46
	Total		50	0	1	6	0	2	59
	N1-specific		0	0	1 *(1.00)*	6 *(1.00)*	0	2 *(1.00)*	8 *(0.13)*
	N2-specific		40 *(0.80)*	0	0	0	0	0	41 *(0.69)*
	Consistent		10 *(0.20)*	0	0	0	0	0	10 *(0.17)*
B (DH populations)			SY	TSW	SN	Pr	O	O/Pr	Sum
AM-DH	LR11	N1	4	3	1	2	2	2	14
		N2	2	3	3	0	0	0	8
		Total	6	6	4	2	2	2	22
		N1-specific	2 *(0.33)*	0	0	2 *(1.00)*	2 *(1.00)*	2 *(1.00)*	8 *(0.36)*
		N2-specific	0	0	2 *(0.50)*	0	0	0	2 *(0.09)*
		Consistent	4 *(0.67)*	6 *(1.00)*	2 *(0.50)*	0	0	0	12 *(0.55)*
	LR12	N1	2	3	2	2	2	0	11
		N2	3	1	2	4	2	0	12
		Total	5	4	4	6	4	0	23
		N1-specific	0	2 *(0.50)*	0	1 *(0.17)*	0	0	3 *(0.13)*
		N2-specific	1 *(0.20)*	0	0	3 *(0.50)*	0	0	4 *(0.17)*
		Consistent	4 *(0.80)*	2 *(0.50)*	4 *(1.00)*	2 *(0.33)*	4 *(1.00)*	0	16 *(0.70)*
	LR13	N1	3	5	1	2	3	1	15
		N2	1	3	1	2	3	0	10
		Total	4	8	2	4	6	1	25
		N1-specific	3 *(0.75)*	2 *(0.25)*	0	1 *(0.25)*	2 *(0.33)*	1 *(1.00)*	9 *(0.36)*
		N2-specific	1 *(0.25)*	0	0	1 *(0.25)*	2 *(0.33)*	0	4 *(0.16)*
		Consistent	0	6 *(0.75)*	2 *(1.00)*	2 *(0.50)*	2 *(0.33)*	0	12 *(0.48)*
	Md11	N1	1	0	1	0	1	0	3
		N2	2	2	2	0	1	0	7
		Total	3	2	3	0	2	0	10
		N1-specific	0	0	0	0	1 *(0.50)*	0	1 *(0.10)*
		N2-specific	1 *(0.33)*	2 *(1.00)*	1 *(0.33)*	0	1 *(0.50)*	0	5 *(0.50)*
		Consistent	2 *(0.67)*	0	2 *(0.67)*	0	0	0	4 *(0.40)*
	Dij13	N1	1	0	0	1	1	2	5
		N2	5	0	0	1	0	0	6
		Total	6	0	0	2	1	2	11
		N1-specific	0	-	-	1 *(0.50)*	1 *(1.00)*	2 *(1.00)*	4 *(0.36)*
		N2-specific	4 *(0.67)*	-	-	1 *(0.50)*	0	0	5 *(0.46)*
		Consistent	2 *(0.33)*	-	-	0	0	0	2 *(0.18)*
DK-DH	LR11	N1	0	2	0	0	3	0	5
		N2	0	1	0	4	4	3	12
		Total	0	3	0	4	7	3	17
		N1-specific	0	1 *(0.33)*	0	0	0	0	1 *(0.06)*
		N2-specific	0	0	0	4 *(1.00)*	1 *(0.14)*	3 *(1.00)*	8 *(0.47)*
		Consistent	0	2 *(0.66)*	0	0	6 *(0.86)*	0	8 *(0.47)*
	Md11	N1	0	1	0	0	0	0	1
		N2	0	2	0	1	4	1	8
		Total	0	3	0	1	4	1	9
		N1-specific	0	0	0	0	0	0	0
		N2-specific	0	1 *(0.33)*	0	1 *(1.00)*	4 *(1.00)*	1 *(1.00)*	7 *(0.78)*
		Consistent	0	2 *(0.67)*	0	0	0	0	2 *(0.22)*

^a(or b)^Number of GWAS-QTL found under the N1 (or N2) condition; ^c^total number of GWAS-QTL; ^d(or e)^number of GWAS-QTL specific to the N1 (or N2) condition with the proportion of the total GWAS-QTL for the corresponding trait indicated in brackets; ^f^number of GWAS-QTL common to the N1 and N2 conditions with the proportion of the total GWAS-QTL for the corresponding trait indicated in brackets. '-',not available. The DTF GWAS-QTL were not included in this table

Legend is as in Table 3A but for LA-QTL

Because the QTL distributions were consistent with the N stress level or were trial-specific, we hypothesized that there was a pattern of QTL based on environmental conditions. We investigated this hypothesis by studying the QTL distribution over clusters of environments.

### Three clusters of environments were defined for each population, and the QTL were mostly cluster-specific

**Fig. 1:**
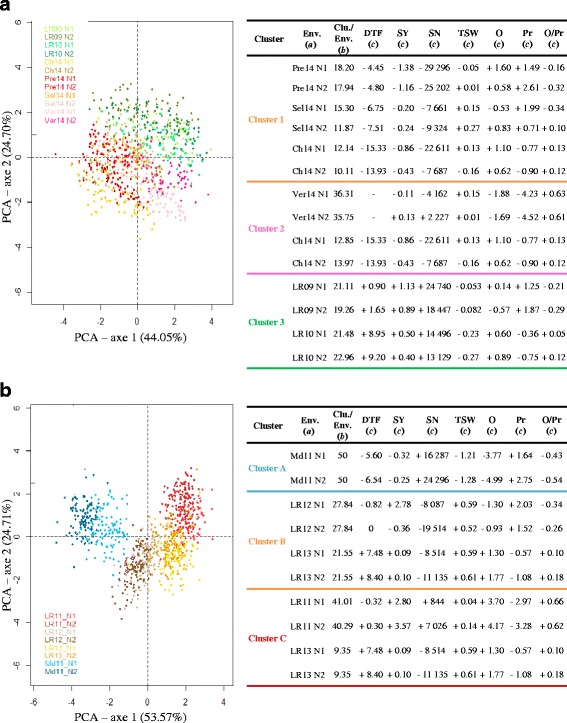
Clustering of the environments based on phenotypic traits. Clustering analysis was performed on the WOSR-69 (a) and AM-DH (b) populations. The projection of the individuals on the first two principal components is presented in the left chart, with individuals colored according to the environment to which they belong. Clusters were defined via hierarchical clustering analysis. Each cluster is characterized by one or several constituent environments (a, Env.), the proportion of each environment in the cluster (b, Clu./Env., expressed as %), and the differences in trait values between the mean of each environment in the cluster and the general mean (c, values given for the different traits evaluated in this study). The trait abbreviations (DTF, SY, SN, TSW, O, Pr, and O/Pr) and units are described in the *Methods* section

**Fig. 2:**
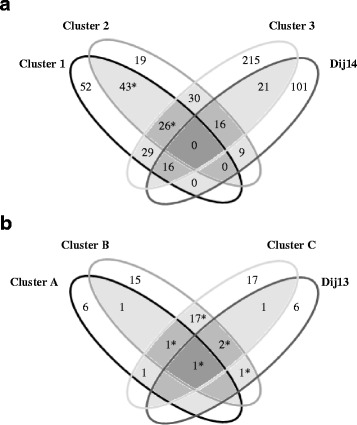
Consistency of the QTL between clusters of environments for the WOSR (A) and AM-DH (B) populations. The QTL were detected for each population and trait using the single-environment model (6) and were counted for each cluster. The numbers in the intersections of the Venn diagrams indicate the numbers of common QTL between the given two, three or four groups. Dij14 (chart a) and Dij13 (chart b) were considered independent groups because they were not included in the clustering. Stars indicate potentially over-estimated numbers due to overlapping trials between clusters 1 and 2 (chart a) or clusters B and C (chart b)

The environments associated with the WOSR-69 population were clustered into three groups, with the two N nutrition conditions for each trial consistently grouped in the same cluster (Fig. 1a). The first cluster (cluster 1) grouped the Pre14, Sel14 and Ch14 trials, which represented 36.14, 27.17 and 22.25 % of the cluster, respectively; cluster 2 grouped the Ver14 and Ch14 trials (72.06 and 26.82 %, respectively); and cluster 3 grouped the LR09 and LR10 trials (40.37 and 44.44 %, respectively). Clusters 1 and 2 were characterized by early flowering (up to 15.33 days below the overall mean DTF value), whereas the LR10 trial in cluster 3 showed the latest flowering time (up to +9.2 days). The yields were lower in clusters 1 and 2 but higher in cluster 3 compared to the mean SY over all environments (from −1.38 to +0.13 t/ha for clusters 1 and 2 and from +0.4 to +1.13 for cluster 3). For the yield components, clusters 1 and 2 were characterized by a lower seed number/m^2^ but higher TSW relative to the mean values. The situation was reversed for cluster 3, with a greater number of smaller seeds. Regarding the seed composition traits, the most striking difference between the three clusters was the low protein content that was obtained for Ver14 in cluster 2 (around −4.5 %). Approximately 67 % of the loci that were detected in the GWAS were specific to one environmental cluster, 23 % were common to two clusters, and 10 % were common to three clusters. No loci were common to all clusters (Fig. 2a).

The environments associated with AM-DH were also clustered into three groups, with the two N nutrition conditions of each trial grouped in the same cluster (Fig. 1b). The first cluster (cluster A) was clearly associated with the Md11 trial, which represented 100 % of the cluster. LR12 was attributed to cluster B (55.68 %) and LR11 to cluster C (81.30 %), whereas LR13 was split between cluster B (43.10 %) and cluster C (18.70 %). Cluster A was characterized by early flowering compared to the mean flowering time over all environments (−5.60 to −6.54 days), a low TSW (−1.21 to −1.28) and a high seed number/m^2^ (+16,287 to +24,296). The opposite trend was observed for cluster B, which was characterized by a high TSW (+0.52 to +0.61) and a low seed number/m^2^ (−8,087 to −19,514). Cluster C was characterized by a higher seed oil content and a lower protein content than the two other clusters. Approximately 65 % of the loci detected in the linkage analyses were specific to one environmental cluster, 30 % were common to two clusters, 4 % were common to three clusters, and one locus was common to all clusters (Fig. 2b).

In conclusion, the loci detected previously via GWAS or linkage analyses were mainly specific to one environmental cluster. However, the QTL of a given cluster were generally distinct between the constitutive trials, suggesting that the QTL × trial interactions predominated over the QTL × cluster interactions.

### The additive genetic control of yield-related traits was assessed by multi-environment model-based genetic analyses

The genetic analyses of the additive genotypic values as estimated for each population using the multi-environment model (1) produced a clear synthetic overview of the consistent QTL for traits related to seed yield and quality. Fifty-one stable QTL were thus identified; all of these QTL were previously detected using a single-trial genetic model (6), confirming their robustness (Additional file 8: Figure S1). Of these, 32 loci were obtained from the WOSR populations, 11 from AM-DH and 8 from DK-DH. The QTL were scattered in all linkage groups except for A7 and C4 (Table 4, Additional file 8: Figure S1). These regions included 27 QTL for seed yield, 7 for flowering time, 6 for seed oil content, 6 for TSW, 2 for seed number/m^2^, 2 for oil/protein ratio, and one for seed protein content.

**Table 4:** Results of the genetic analyses performed on the WOSR (A) or DH (B) populations for [a] the genotypic estimates extracted from the multi-environment model (${\boldsymbol{P}}_{\boldsymbol{i}\boldsymbol{jklm}} = \boldsymbol{\mu} +\underline {{\boldsymbol{G}}_{\boldsymbol{i}}} + {\boldsymbol{N}}_{\boldsymbol{j}} + \underline {{\boldsymbol{T}}_{\boldsymbol{l}}}+\underline {{\boldsymbol{T}}_{\boldsymbol{l}}\left({\boldsymbol{R}}_{\boldsymbol{k}}\right)} + \underline {{\boldsymbol{G}}_{\boldsymbol{i}}\times {\boldsymbol{N}}_{\boldsymbol{j}}}+\underline {{\boldsymbol{G}}_{\boldsymbol{i}} \times {\boldsymbol{T}}_{\boldsymbol{l}}} + \underline {{\boldsymbol{G}}_{\boldsymbol{i}}\times {\boldsymbol{N}}_{\boldsymbol{j}}\times {\boldsymbol{T}}_{\boldsymbol{l}}} + \underline {{\boldsymbol{e}}_{\boldsymbol{i}\boldsymbol{jklm}}}$ (model 1)) and [b] the ecovalence values calculated using the G × N or G × T model (${\boldsymbol{W}}_{\boldsymbol{i}} = {\displaystyle \sum_{\boldsymbol{i}}}\left({\boldsymbol{Y}}_{\boldsymbol{i}\boldsymbol{j}}-{\boldsymbol{Y}}_{\boldsymbol{i}.}-{\boldsymbol{Y}}_{.\boldsymbol{j}}+{\boldsymbol{Y}}_{..}\ \right)2$ (model 8))

A (WOSR populations)												
Model (a)	Trait	LG	Marker (b)	Position (cM) (c)	Position (bp) (d)	MAF (e)	FDR (f)	R^2^ (g)	CI (cM) (h)	CI (bp) (i)	Favorable allele (j)	Additivity (k)
G [a]	SY	A1	Bn-A01-p1000115	3.0	617,658	0.18	0.08	0.13	1.73	234,187	T	0.25
			Bn-A01-p5480514	36.4	5,042,546	0.43	0.10	0.13	1.50	158,442	G	0.18
			Bn-A01-p20990218	65.3	17,795,523	0.21	0.10	0.12	0.30	370,421	C	0.23
			Bn-A01-p24697185	74.6	20,465,114	0.40	0.10	0.12	5.70	184,781	G	0.20
		A3	Bn-A03-p766322	1.1	634,722	0.39	0.14	0.11	4.70	400,479	A	0.20
			Bn-C3-p15411996	64.8	9,973,043	0.10	0.13	0.12	72.10	315,568	T	0.27
		A4	Bn-A04-p11510797	22.1	12,567,132	0.10	0.02	0.21	0.65	323,361	A	0.39
		A5	Bn-A05-p4108818	32.1	3,986,210	0.32	0.15	0.11	6.00	608,673	A	0.20
			Bn-A05-p6897871	48.8	6,348,806	0.07	0.03	0.17	7.10	1,584,822	A	0.40
			Bn-A05-p23166612	99.8	21,325,804	0.19	0.02	0.18	6.15	2,427,395	C	0.30
		A8	Bn-A08-p14156050	17.9	11,851,743	0.42	0.15	0.11	2.30	390,921	C	0.19
			Bn-A08-p16125811	28.2	13,582,310	0.06	0.15	0.11	3.35	370,297	G	0.31
		A9	Bn-A09-p3024539	20	2,944,505	0.49	0.02	0.22	32.53	5,529,615	T	0.28
			Bn-A08-p9226756	65.5	14,301,186	0.09	0.15	0.11	0.00	1,533,647	T	0.29
		A10	Bn-A10-p9052583	13.5	10,444,212	0.10	0.12	0.12	6.80	1,679,566	A	0.29
		C1	Bn-C1-p5199797	31.5	5,036,240	0.31	0.09	0.13	0.00	410,741	A	0.21
			Bn-C1-p37170472	77.4	34,962,509	0.17	0.09	0.13	0.00	1,141,921	A	0.23
		C2	Bn-C2-p50647994	125.4	44,768,073	0.40	0.06	0.15	39.00	5,044,117	C	0.22
		C5	Bn-C5-p45854218	117.0	42,566,107	0.29	0.07	0.14	1.50	230,043	A	0.20
		C6	Bn-C6-p19300068	42.9	18,948,227	0.12	0.06	0.14	8.20	1,201,949	G	0.30
			Bn-C6-p8469110	65.9	28,380,973	0.37	0.15	0.11	9.50	5,946,818	C	0.21
		C7	Bn-C7-p32630456	41.0	32,902,306	0.22	0.02	0.21	0.70	3,328,120	T	0.31
			Bn-C7-p37740616	57.8	34,843,632	0.22	0.02	0.20	3.68	1,113,132	A	0.32
		C8	Bn-C8-p21614432	31.8	20,898,335	0.28	0.11	0.12	6.40	545,619	G	0.21
		C9	Bn-C9-p2006237	16.3	872,443	0.49	0.03	0.17	12.80	3,275,350	G	0.26
	O	A5	Bn-A02-p26656981	103.2	22,149,643	0.20	0.12	0.21	2.86	976,652	G	0.84
		A9	Bn-A09-p1989556	15.6	1,964,619	0.33	0.12	0.27	12.60	2,168,340	A	0.84
			Bn-A09-p6950255	50.4	5,891,565	0.37	0.12	0.24	0.55	329,763	A	0.74
		C1	Bn-C1-p5199797	31.5	5,036,240	0.31	0.12	0.23	3.14	1,808,094	A	0.78
		C7	Bn-C7-p36749441	53.6	33,959,826	0.20	0.12	0.23	10.22	3,157,954	A	0.93
			Bn-C7-p38504198	59.9	35,328,335	0.32	0.14	0.20	2.75	651,737	T	0.73
	O/Pr	A9	Bn-A09-p3051349	20.3	2,971,796	0.46	0.13	0.24	4.80	745,210	G	0.13
G × N [b]	O	A5	Bn-A02-p26656981	103.2	22,149,643	0.20	0.12	0.21	2.86	976,653	G	0.32
		A9	Bn-A09-p1989556	15.6	1,964,619	0.33	0.12	0.27	12.60	2,168,341	A	0.32
			Bn-A09-p6950255	50.4	5,891,565	0.37	0.12	0.24	0.55	329,762	A	0.28
		C1	Bn-C1-p5195180	31.5	5,031,632	0.31	0.12	0.23	3.14	1,771,001	T	0.30
		C7	Bn-C7-p35762591	51.0	33,046,261	0.20	0.12	0.23	10.22	3,825,530	G	0.35
G × T [b]	SY	A1	Bn-A01-p3005793	22.7	2,717,050	0.12	0.15	0.20	25.00	467,612	T	1.42
			Bn-A01-p19929280	62.9	16,718,048	0.07	0.15	0.19	1.20	953,751	G	1.67
			Bn-A01-p23321507	70.1	19,192,316	0.06	0.06	0.31	5.40	2,053,778	C	2.38
		A3	Bn-A03-p3795299	31.7	3,348,503	0.07	0.15	0.20	1.00	142,079	A	1.69
			Bn-A03-p19897251	108	18,786,539	0.08	0.15	0.20	1.80	309,561	A	1.71
		A4	Bn-A04-p8348806	11.2	9,654,868	0.06	0.06	0.30	0.30	290,373	T	2.52
		A7	Bn-A07-p10750190	27.6	11,956,021	0.08	0.15	0.23	13.35	2,016,127	A	1.80
			Bn-A07-p13485722	45.2	15,490,316	0.07	0.09	0.26	0.90	179,009	T	1.95
		C6	Bn-C6-p10394225	64.7	26,530,886	0.07	0.15	0.21	3.00	2,341,718	C	1.73
			Bn-C6-p4142963	74.6	32,815,121	0.13	0.15	0.19	5.58	855,273	A	1.30
	O	A3	Bn-A03-p3857752	32.2	3,414,578	0.12	0.03	0.36	1.50	243,919	C	407.71
			Bn-A03-p19897251	108	18,786,539	0.08	0.00	0.56	8.20	1,991,427	A	646.30
		A5	Bn-A05-p5372015	39.9	5,159,907	0.06	0.05	0.32	9.80	1,033,840	T	527.86
			Bn-A05-p23876386	104.6	22,847,000	0.17	0.13	0.28	2.60	397,014	T	333.29
		A8	Bn-A08-p10270998	5.3	8,220,334	0.06	0.06	0.31	14.40	7,267,401	A	518.40
			Bn-A08-p14375712	20.3	12,062,255	0.07	0.15	0.27	0.90	299,176	G	435.05
		C4	Bn-C4-p1583238	7.5	1,269,444	0.12	0.04	0.34	0.00	543	G	406.32
			Bn-C4-p9031482	47.8	8,359,826	0.06	0.05	0.32	3.30	643,168	C	527.86
	Pr	A3	Bn-A03-p3862928	32.2	3,422,213	0.10	0.06	0.34	1.40	73,710	C	96.42
			Bn-A03-p11364656	68.3	10,465,900	0.07	0.11	0.27	0.60	120,617	G	99.10
			Bn-A03-p19897251	108	18,786,539	0.08	0.02	0.45	6.10	1,665,096	A	126.30
	O/Pr	A1	Bn-A01-p1409723	6.7	1,006,909	0.07	0.10	0.29	1.24	148,550	A	2.21
			Bn-A01-p3378332	24.8	3,044,540	0.10	0.15	0.25	0.00	131,684	A	1.84
		A3	Bn-A03-p3851195	32.2	3,406,943	0.12	0.08	0.31	2.00	212,147	T	1.85
			Bn-A03-p11364656	68.3	10,475,189	0.07	0.12	0.28	0.90	223,645	G	2.16
			Bn-A03-p19897251	108	18,786,539	0.08	0.00	0.55	8.20	1,991,427	A	3.03
		A7	Bn-A07-p16965943	67.1	18,885,532	0.06	0.07	0.35	2.00	190,634	T	2.71
B: (DH populations)												
Model(l)	Population	Trait	LG	Marker (m)	Position (cM) (n)	Position (bp) (o)	LOD (p)	R^2^ (q)	CI (cM) (r)	CI (bp) (s)	Favorable allele (t)	Additivity (u)
G [a]	AM-DH	DTF	A1	Bn-A01-p821328	0.9	449,990	10.48	0.19	1.8	157,286	Aviso	1.67
			A2	Bn-A02-p10621504	31.6	7,486,332	10.73	0.20	2	1,268,217	Aviso	1.34
			C2	Bn-C2-p2374956	22	-	12.51	0.24	4.5	2,829,279	Aviso	1.38
			C6	Bn-C6-p4847689	54	32,036,565	12.08	0.23	7.4	4,825,658	Montego	1.58
		SY	A5	BS005820	30.7	3,536,588	7.2	0.21	5.8	577,487	Montego	0.92
			C3	Bn-C3-p1719357	8.2	1,592,799	5.72	0.16	5.1	749,919	Aviso	0.84
		SN	A1	Bn-A01-p2884934	22.4	2,388,770	4.76	0.15	19.6	2,415,980	Montego	1513.74
			A5	BS006984	44.4	8,910,408	4.43	0.14	39.8	17,132,188	Montego	1794.32
		TSW	C3	BS010983	136.7	51,428,020	3.57	0.11	20	7,091,270	Aviso	0.05
			C4	BS009060	0	834,581	3.66	0.11	22.4	11,856,417	Aviso	0.07
			C5	Bn-C5-p41401139	66.3	38,568,003	4.15	0.12	10	3,714,187	Montego	0.06
	DK-DH	DTF	A2	BS006299	44	375,658	12.87	0.28	7.4	3,335,785	Express	1.67
			A10	BS010793	50	2,137,761	6.59	0.12	3.8	651,272	Express	1.18
			C6	Bn-C6-p8468434	63.7	28,381,646	16.92	0.41	1.6	82,072	Tenor	2.32
		TSW	A5	BS038657	83.7	-	3.57	0.12	18	1,623,621	Express	0.05
			A6	Bn-A06-p3184395	37.1	3,059,935	4.98	0.17	11	591,264	Tenor	0.06
			C2	Bn-C2-p7577094	12	-	5.1	0.17	10	8,848,115	Tenor	0.07
		O/Pr	C2	Bn-Scaffold00285-p518297	42.9	39,511,111	3.45	0.17	15.7	7,299,924	Express	0.01
		Pr	C2	Bn-Scaffold00285-p518297	42.9	39,511,111	4.07	0.20	24	7,170,032	Tenor	0.11
G × N [b]	AM-DH	SN	A1	Bn-A01-p2841043	22.4	2,350,106	4.93	0.16	15.6	936,796	Montego	9.0.10^5^
			A5	Bn-A05-p15236669	45.3	11,713,855	4.79	0.16	39.8	8,839,936	Montego	8.8.10^5^
		TSW	A1	Bn-A01-p2841043	22.4	2,350,106	3.89	0.07	11.8	1,358,912	Aviso	0.01
			A4	BS009059	0	834,628	8.2	0.17	8.9	5,557,003	Aviso	0.01
			A7	Bn-A07-p10635069	21.7	11,816,784	3.83	0.07	14.3	2,286,113	Aviso	0.01
			A9	Bn-A09-p32257776	109.2	30,105,456	5.09	0.10	10	36,978	Montego	0.01
			C3	Bn-C3-p55422785	136.7	51,342,596	5.82	0.11	5.1	1,520,491	Aviso	0.01
			C5	Bn-C5-p41401139	66.3	38,568,003	5.91	0.12	4	1,265,089	Montego	0.01
		O	C1	Bn-C1-p7004482	44.6	6,770,122	3.56	0.14	22	4,433,568	Montego	0.35
		Pr	A1	BS010224	69.5	19,925,267	3.49	0.14	48	18,210,785	Aviso	0.28
		O/Pr	A2	Bn-A02-p26398002	74.4	24,112,871	3.66	0.13	16	1,091,767	Montego	1.82.10^−3^
			C3	Bn-C3-p579321	1	520,340	3.81	0.14	2.8	441,719	Aviso	1.90.10^−3^
	DK-DH	DTF	A2	Bn-A02-p9506103	43.7	6,320,247	14.1	0.30	4	2,927,770	Express	14.1
			A10	BS010815	44.9	15,547,512	5.63	0.09	8	651,272	Express	5.63
			C6	Bn-C6-p8468434	63.7	28,381,646	17.41	0.41	1.3	790,491	Express	17.41
		TSW	A5	Bn-A05-p21375514	83.7	19,544,037	4.14	0.14	20	1,334,758	Express	4.14
			A6	Bn-A06-p3184395	37.1	3,059,935	4.57	0.16	9.7	162,861	Express	4.57
			C2	Bn-Scaffold01089-p23965	11.5	6,472,037	4.47	0.15	12.3	2,436,439	Express	4.47
		Pr	C2	Bn-Scaffold00285-p518297	42.9	39,511,111	4.35	0.21	15.6	7,299,924	Express	4.35
		O/Pr	A6	Bn-A06-p18634396	85	19,998,443	4.61	0.14	15.3	1,358,110	Express	1.88.10^−5^
			A8	Bn-A08-p14799839	21.3	12,362,293	3.77	0.11	10	999,231	Tenor	1.93.10^−5^
			C2	Bn-C3-p24936161	39.5	36,232,623	6.23	0.20	7	1,632,362	Express	2.18.10^−5^
G × T [b]	AM-DH	DTF	A1	Bn-A01-p1150857	5.2	754,478	7.55	0.13	7	537,061	Aviso	55
			A2	Bn-A02-p7892272	31.6	4,878,455	10.63	0.20	3.6	2,645,488	Aviso	59.93
			C2	Bn-C2-p2054626	19	246,359	10.27	0.19	6.8	202,734	Aviso	49.08
			C6	Bn-C6-p8905311	47.2	27,985,250	9.79	0.18	7.3	298,022	Montego	48.73
		SN	A1	Bn-A01-p2069488	13.3	1,561,728	4.74	0.19	19.2	1,921,336	Montego	2.34.10^8^
		Pr	A1	Bn-A01-p2538180	17.3	2,035,020	3.12	0.11	16.8	1,601,066	Aviso	2.6
			A9	Bn-C9-p17562068	54.4	9,942,680	4.49	0.16	15.4	17,290,961	Aviso	3.28
	DK-DH	DTF	A2	Bn-A02-p9506103	43.7	6,320,247	13.09	0.29	4.2	2,152,190	Express	13.09
			A10	A10_BS010815	44.9	15,547,512	5.14	0.09	7.8	651,272	Express	41.43
			C6	Bn-C6-p8468434	63.7	28,381,646	16.43	0.40	1.3	790,491	Tenor	84.49
		TSW	C2	Bn-C2-p17324647	19.1	13,977,345	4.92	0.24	11.5	16,386,686	Tenor	0.34
		Pr	C2	Bn-Scaffold00285-p518297	42.9	39,511,111	4.01	0.20	15.6	7,299,924	Tenor	6.7
		O/Pr	C2	Bn-Scaffold00285-p518297	42.9	39,511,111	3.26	0.16	13.9	6,363,073	Express	0.24

(a) For the model considered in [a], genotypic estimates were extracted from multi-environment model (1), and for the model considered in [b], ecovalence values were calculated using G × N or G × T model (8)

(b) SNP at the most significant position of the QTL

(c) Genetic position of the most significant marker on the WOSR map (in cM)

(d) Physical position of the most significant marker within the physical sequence of *B. napus* (in bp)

(e) Minor allele frequency

(f) False discovery rate

(g) Phenotypic variance explained by the most significant SNP

(h) Confidence interval of the QTL on the WOSR genetic map (in cM)

(1) Confidence interval of the QTL within the genomic sequence (in bp)

(j) Favorable allele at the most significant SNP

(k) Allelic effect of the favorable allele at the most significant position

(l) Model considered (as in Table 4A)

(m) SNP at the most significant position of the QTL

(n) Genetic position of the most significant marker on the WOSR map (in cM)

(o) Physical position of the most significant marker within the physical sequence of *B. napus* (in bp)

(p) Log of likelihood of the genetic model for the tested QTL

(q) Phenotypic variance explained by the most significant SNP

(r) Confidence interval of the QTL on the WOSR genetic map (in cM)

(s) Confidence interval of the QTL within the genomic sequence (in bp)

(t) Favorable allele at the most significant SNP

(u) Allelic effect of the favorable allele at the most significant position

Nine of the seed yield QTL showed putative colocalizations with loci controlling other traits, including flowering time (A1 and C6), seed weight (A4), seed number/m^2^ (A5), oil content (A5, A9, C1 and C7), and protein content (C2) (Additional file 8: Figure S1).

Most of the QTL that were detected with model (1) were specific to a given population. Indeed, only three genomic regions were consistent between two different populations, including two loci controlling flowering time in the A2 and C6 linkage groups detected in the AM-DH and DK-DH populations and one locus for seed yield in A5 detected in the WOSR and AM-DH populations.

### Exploration of the loci contributing to the G × N and/or G × T interactions according to the ecovalence traits

The genetic analyses performed using the ecovalence values that were calculated with the G × N or G × T models revealed the loci contributing to the interactions of the genotypes with the N nutrition condition or the trial, respectively. These analyses revealed 27 and 40 QTL controlling the G × N and G × T interactions, respectively, for the three populations (Table 4, Additional file 8: Figure S1).

Five, 12 and 10 G × N QTL were detected in the WOSR, AM-DH and DK-DH populations, respectively. Eighteen of these QTL colocalized with QTL that were detected using the multi-environment model (1), suggesting that these loci were involved in both the additive and the G × N components (Additional file 8: Figure S1). Some of the loci were previously found to be environment-specific based on the single-environment model (6). For instance, a QTL for TSW in the A5 LG, which was specific to the N2 condition based on the single-environment model, colocalized with a G × N QTL. On the other hand, five QTL for DTF, seed number/m^2^ and oil content in A1, A5, C6 and C7, which were detected under both N nutrition conditions using the single-environment model, colocalized with G × N QTL. These colocalizations may therefore reflect a modulation of the effects of the QTL between N nutrition conditions rather than the presence or absence of a relationship between treatments. In addition, nine G × N QTL did not colocalize with any other QTL and formed specific N-interactive loci (Additional file 8: Figure S1). These loci corresponded to N-specific QTL that were detected using the single-environment model (6) (data not shown).

Twenty-seven, 7 and 6 G × T QTL were detected for the WOSR, AM-DH and DK-DH populations, respectively. Fifteen of these QTL colocalized with QTL that had previously been detected using the multi-environment model. In addition, four QTL for seed yield and oil content in the A1, A4 and A5 LG that were previously found to be trial-specific based on the single-environment model colocalized with G × T QTL. Five QTL for seed yield and oil content in the A5, A9, C1 and C9 LG that were consistent across trials according to the single-environment model did not colocalize with any G × T QTL, confirming their stability. On the other hand, six QTL for DTF in the A1, A2, C2 and C6 LG that were detected across at least five trials colocalized with G × T QTL (Additional file 8: Figure S1). Twenty-four of these QTL did not colocalize with any of the fifty-one QTL detected using the multi-environment model and instead represented specific trial-interactive loci (Additional file 8: Figure S1).

In summary, colocalizations of QTL detected using the multi-environment model and genomic regions contributing to the G × N and G × T interactions pinpointed loci with additive effects modulated by the N fertilization condition (11 QTL), the trial (9 QTL) or both (6 QTL). Nine and twenty-five loci were specific to the G × N and G × T interactions, respectively, but no region specific to both interactions was detected.

### Structural organization of the key loci controlling seed yield in the *B. napus* genome

To gain insight into the genomic organization of the main regions controlling seed yield, we first determined the homoeologous relationships between predicted genes within the QTL that were detected using the multi-environment model according to the *B. napus* Darmor-*bzh* reference genome [30]. We focused only on the forty-two QTL that were associated with seed yield-related factors *per se*, including flowering time, seed yield, seed number/m^2^, and TSW.

**Fig. 3:**
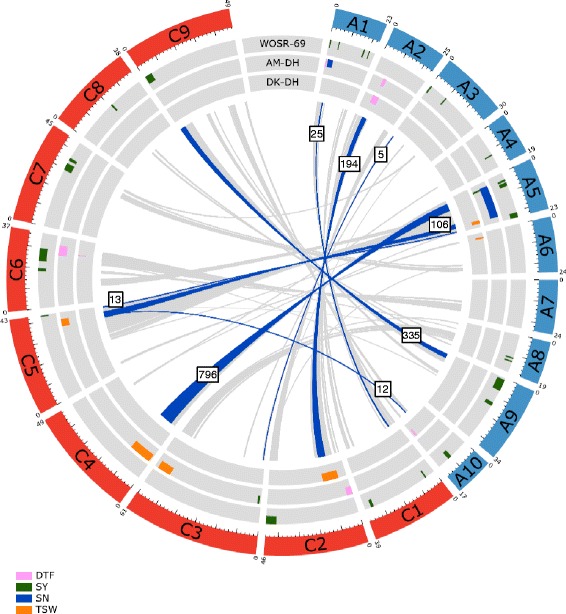
Circos diagram showing the homoeologous relationships between genes present within the main regions for yield-related traits. The 19 *B. napus* chromosomes (2*n* = 4*x* = 38; AACC) belonging to the A and C sub-genomes are represented in blue and red, respectively. The physical size of each chromosome (in Mbp) is indicated above the chromosome, and a ruler is drawn below, with larger and smaller tick marks every 10 and 2 Mbp, respectively. The forty-two QTL seed yield-related traits detected using model (1) (${\boldsymbol{P}}_{\boldsymbol{i}\boldsymbol{jklm}} = \boldsymbol{\mu} +\underline {{\boldsymbol{G}}_{\boldsymbol{i}}} + {\boldsymbol{N}}_{\boldsymbol{j}} + \underline {{\boldsymbol{T}}_{\boldsymbol{l}}}+\underline {{\boldsymbol{T}}_{\boldsymbol{l}}\left({\boldsymbol{R}}_{\boldsymbol{k}}\right)} + \underline {{\boldsymbol{G}}_{\boldsymbol{i}}\times {\boldsymbol{N}}_{\boldsymbol{j}}}+\underline {{\boldsymbol{G}}_{\boldsymbol{i}} \times {\boldsymbol{T}}_{\boldsymbol{l}}} + \underline {{\boldsymbol{G}}_{\boldsymbol{i}}\times {\boldsymbol{N}}_{\boldsymbol{j}}\times {\boldsymbol{T}}_{\boldsymbol{l}}} + \underline {{\boldsymbol{e}}_{\boldsymbol{i}\boldsymbol{jklm}}}\Big)$ are represented by bars on three runways, depending on the population in which the QTL was detected (WOSR-69, AM-DH or DK-DH) according to the following color code: flowering time (DTF) in *pink*; seed yield (SY) in *green*; seed number/m^2^ (SN) in *blue*; and thousand-seed weight (TSW) in *orange*. Each pair of homoeologous genes is represented by a line colored according to the following code: in *gray*, the homoeologous genes between QTL and regions carrying no other QTL; in *blue*, the homoeologous genes between QTL detected using model (1). The numbers of homoeologous genes present in each pair of homoeologous regions are indicated in the boxes

Homoeologous relationships were observed between twelve QTL in the A genome and eight QTL in the C genome (Fig. 3). Pairs of homoeologous QTL controlling the same traits were identified for seed yield (A3-C3; A5-C5; A9-C9) and TSW (A5-C5), as well as homoeologous QTL controlling different traits, such as DTF - TSW (A2-C2), seed yield - seed number/m^2^ (A1-C1), seed yield - TSW (A5-C4; A10-C5), and seed number/m^2^ - TSW (A5- C4/C5) (Fig. 3).

In addition, for each pair of homoeologous QTL controlling the same trait that were detected in the WOSR-69 population, the frequency of the favorable allele in the most significant positions of the QTL was compared between copies. Although two homoeologous QTL for seed yield in A9 and C9 showed similar frequencies of the favorable allele in the WOSR-69 population (A9: 0.48; C9: 0.49), the frequency of the favorable allele was unbalanced for the QTL pair for seed yield in the A5 and C5 LG (A5: 0.19; C5: 0.71). This result suggests the differential evolution of QTL copies between the two sub-genomes for some of the homoeologous regions.

Finally, each pair of QTL showed 5 to 796 homoeologous genes (Fig. 3) that may be considered candidate genes for the corresponding traits.

## Discussion

### The N nutrition level may not be the only limiting factor

Although several precautions were taken during cropping to limit the amount of mineral N in the soil in the experimental plots, the N stresses that were applied in the different trials were often moderate to absent based on the NNI values as measured during the bolting stage. This finding suggests that low-N nutrition conditions were not limiting in most trials, probably due to the highly favorable environmental conditions, such as mild climate, and/or soil features, which promote substrate mineralization. Nevertheless, a difference in the N nutrition level was observed in many trials, even for those showing a low stress level, which was reflected in the difference in seed yield between the two N fertilization conditions (approximately 0.50 t/ha).

This observation was confirmed by highly significant N effects on all of the traits in the mixed model analyses. In contrast, low G × N interactions were found in most of the population × trial combinations, and a majority of the QTL were stable between the two N conditions, with fewer QTL detected under limited N fertilization conditions than under the sub-optimal N nutrition conditions. These findings are consistent with previous results of few G × N [52, 53] or QTL × N [17] interactions in rapeseed but contradicts the observations of Nyikako *et al.* [54], who detected G × N interactions. The identification of G × N interactions primarily depends on the environments tested as well as on the genetic diversity of the populations studied (a high genetic variance may conceal the relative G × N effects).

At least five trials were set up to evaluate each population in our study. Strong trial (T) and G × T effects were found for most of the studied traits, probably due to the specific environmental conditions in each trial. For example, water stress was observed in the Md11 trials at the flowering stage, consistent with the results that the corresponding environments clustered independently and that the heritability values were lower than in the other environments. Thus, dense networks of trials as well as a precise agronomic characterization of the environments are needed for consistent phenotypic evaluation. Indeed, many QTL × T interactions were observed, with more than 50 % of the QTL being specific to a single cluster of environments and even to a single trial. In bread wheat, Kuchel *et al.* [55] reported that approximately one quarter of the G × E interactions could be explained by interaction of the QTL with climatic cofactors. El-Soda *et al.* [56] reported that most QTL displayed QTL × E interactions in Arabidopsis, although their effects on the trait values can vary (*e.g.*, opposing effects, differences in intensity). Similarly, QTL × E interactions have also been found in apples [57], cotton [58], rice [59], and wheat [60], whereas a high consistency of QTL between environments was reported in maize [61]. These discrepancies may be explained by the heritability of the traits across the environments or by the environments tested.

### Refining the genetic architecture of complex traits

How to increase the precision of QTL detection is a central question in genetic analyses. Two ways are addressed in the present work. First, the genetic analyses of additive effects using multi-environment models and second, the combination of the results from complementary approaches, such as linkage analyses and GWAS.

In the GWAS and linkage analyses that were performed on each environment (model 6), 1,130 loci that controlled one or several of the nine traits under study were detected, and these loci were distributed over the 19 *B. napus* linkage groups. The complexity of the QTL architecture for seed yield and the difficulty of handling massive QTL sets were also assessed by Shi *et al.* [62], who detected 870 QTL for seed yield in two rapeseed populations grown in ten environments. This information could be used to assess the impact of the trials and the environmental clusters on the genetic architecture of seed yield traits. However, the large number of trial-specific QTL hindered interpretation of the results. Multi-environment QTL analyses were suggested to overcome the impact of QTL × E interactions because these analyses are based on multiple environmental datasets and thus lead to more robust QTL than single-environment analyses [63]. In our study, the set of QTL detected using the single-environment model could be reduced to fifty-one robust QTL using the multi-environment model (1), and this restricted dataset allowed for a more synthetic overview of the major regions involved in the control of seed yield traits.

Combining linkage analyses and GWAS has been successfully reported for several species, including rapeseed [9, 64]. In our study, we showed that a small proportion of the QTL were consistent between population structures (DH *versus* WOSR populations), underlying the complementarity of the two approaches to decipher the genetic architecture of complex traits. Another method that has been successfully tested in maize [65] integrates both GWAS and linkage analyses into a single analysis (integrated mapping, or joint mapping), allowing several genetic parameters to be tested simultaneously. The development of new types of mapping populations calibrated to perform joint linkage analyses-GWAS, such as nested association mapping (NAM) [66] or multi-parent advanced generation inter-cross (MAGIC) populations [67], is ongoing in several plant species, including rapeseed, to optimize both the power of QTL detection and the diversity of the tested alleles.

### Genetic analyses of the ecovalence values revealed the QTL × N and QTL × T interactions

In most studies in which the genetic architecture of a trait is analyzed under several treatments, direct comparisons of QTL or analyses of the differences in their effects between treatments are the most common methods employed. Genetic analyses with stability parameters, such as ecovalence, are another way to identify genomic regions involved in G × N or G × T interactions. This type of genetic analysis has previously been performed on barley [28], in which the colocalization of ecovalence-related QTL with earliness genes was determined. In our study, we found colocalizations between QTL for seed yield traits detected using the multi-environment model (1) and QTL for G × N or G × T interactions. These results suggest that these regions showed allelic variations conferring adaptation to specific environments. In addition, 9 and 25 QTL specific to G × N and G × T interactions, respectively, were detected, and these QTL represent candidate regions for increasing NUE and for promoting adaptation to different environmental conditions in rapeseed.

### Genomic tools provide clues regarding the organization and gene content of seed yield-related loci

The newly released *B. napus* genomic sequence [30] has provided new directions for QTL analyses in terms of gene content. Therefore, we were able to compare the gene repertoire in the forty-eight yield-related QTL and to assess their homoeologous relationships within the whole rapeseed genome. Indeed, several other studies previously described the occurrence of duplicated QTL for glucosinolate content or stem canker in rapeseed [68], and these findings are consistent with the allopolyploid origin of the *B. napus* genome [69]. Thus, we aimed to address whether homoeologous regions control seed yield-related traits. Our results showed that twenty of the QTL displayed homoeologous genes within the confidence intervals of at least one QTL controlling a related trait.

The homoeologous genes covered by QTL for the same traits may be considered as promising candidates. However, due to the large confidence intervals obtained in our study, such genes remain numerous (up to 796 genes in our study) and difficult to analyze. One possible way to decipher trends of potential functions underlying these regions would be to use the Gene Ontology (GO) network [70]. Recently, Bargsten *et al.* [71] proposed a method of candidate gene prioritization for the analysis of 1,591 QTL regions found in rice associated with 231 different traits. They compared the occurrence of the biological functions of the genes found in the regions associated with one trait to the rest of the genome and retained the genes displaying significant over-representation of a given function. This method led to a ten-fold decrease in the number of putative candidate genes. Combining high-throughput gene annotations with QTL data could therefore lead to the elucidation of the underlying functions of the QTL.

## Conclusions

We found that under our environmental conditions, the effect of the trial was greater than the effect of N nutrition level on seed yield-related traits. This result paves the way for the development and use of new indicators of plant and environmental status to assess the most limiting factors during sensitive stages of yield production. Nevertheless, in the present study, fifty-one novel main regions involved in the yield of rapeseed were identified and were stable across populations and environments. It appeared that these regions gathered QTL for different traits related to yield and its components, suggesting possible pleiotropic effects. To go further, an analysis of the gene content and the corresponding functions of the related genes may provide evidence indicating the molecular determinants of seed yield and yield-related traits in rapeseed.

## Additional files

Additional file 1: Table S1. Accessions of the WOSR populations. (XLSX 13 kb) Additional file 2: Table S2. Linkage disequilibrium decay within the WOSR populations. (XLSX 17 kb) Additional file 3: Table S3. Trial designs and crop management strategies. (XLSX 14 kb) Additional file 4: Table S4. Results of the mixed linear models applied to each population × trial combination (models (3) and (4)). (XLSX 22 kb) Additional file 5: Table S5. Phenotypic and genetic correlations between the traits measured under N1 and N2 conditions for the different populations. (XLSX 15 kb) Additional file 6: Table S6. Results of the GWAS for the WOSR populations for each trial × N × trait combination. (XLSX 150 kb) Additional file 7: Table S6. Results of the linkage analyses for the DH populations for each trial × N × trait combination. (XLSX 38 kb) Additional file 8: Figure S1. QTL mapping onto the WOSR map. (PPTX 272 kb)

## Abbreviations

AM-DH: Aviso × Montego DH population
Ch14: Châteauroux trial in the 2013–2014 season
DH: Doubled haploid
Dij13: Dijon trial in the 2012–2013 season
Dij14: Dijon trial in the 2013–2014 season
DK-DH: Tenor × Express DH population
DTF: Days to flowering
FDR: False discovery rate
GxE: Genotype by environment interaction
GxN: Genotype by nitrogen interaction
GxT: Genotype by trial interaction
GO: Gene Ontology
GWAS: Genome-wide association study
K matrix: Identity-by-state kinship matrix
LA: Linkage analysis
LD: Linkage disequilibrium
LG: Linkage Group
LR09: Le Rheu trial in the 2008–2009 season
LR10: Le Rheu trial in the 2009–2010 season
LR11: Le Rheu trial in the 2010–2011 season
LR12: Le Rheu trial in the 2011–2012 season
LR13: Le Rheu trial in the 2012–2013 season
MAF: Minor allele frequency
MAGIC: Multi-parent Advanced Generation Inter-Cross
Md11: Mondonville trial in the 2010–2011 season
MQM: Multiple QTL Mapping
N: Nitrogen
N1: Low N conditions
N2: Optimal N conditions
NAM: Nested Association Mapping
NNI: Nitrogen nutrition index
NUE: Nitrogen use efficiency
O: Seed oil content
O/Pr: Seed oil content/seed protein content ratio
PCA: Principal Component Analysis
Pr: Seed protein content
Pre14: Prémesques trial in the 2013–2014 season
QTL: Quantitative trait locus
Sel14: Selommes trial in the 2013–2014 season
SN: Seed number per m^2^
SNP: Single nucleotide polymorphism
SSR: Simple sequence repeat
SY: Seed yield
TSW: Thousand-seed weight
Ver14: Verpillères trial in the 2013–2014 season
WOSR: Winter oilseed rape
WOSR-92: Population of 92 WOSR accessions for GWAS
WOSR-69: Subset of the WOSR-92
