# Supplementary material for: Genetic basis of nitrogen use efficiency and yield stability across environments in winter rapeseed
Source: BMC Genet. 2016 Sep 15;17:131. doi: 10.1186/s12863-016-0432-z (PMC5024496; doi:10.1186/s12863-016-0432-z)
Supplement: Additional file 8: Figure S1. — QTL mapping onto the WOSR map. (PPTX 272 kb) [file 12863_2016_432_MOESM8_ESM.pptx]

## Slide 1
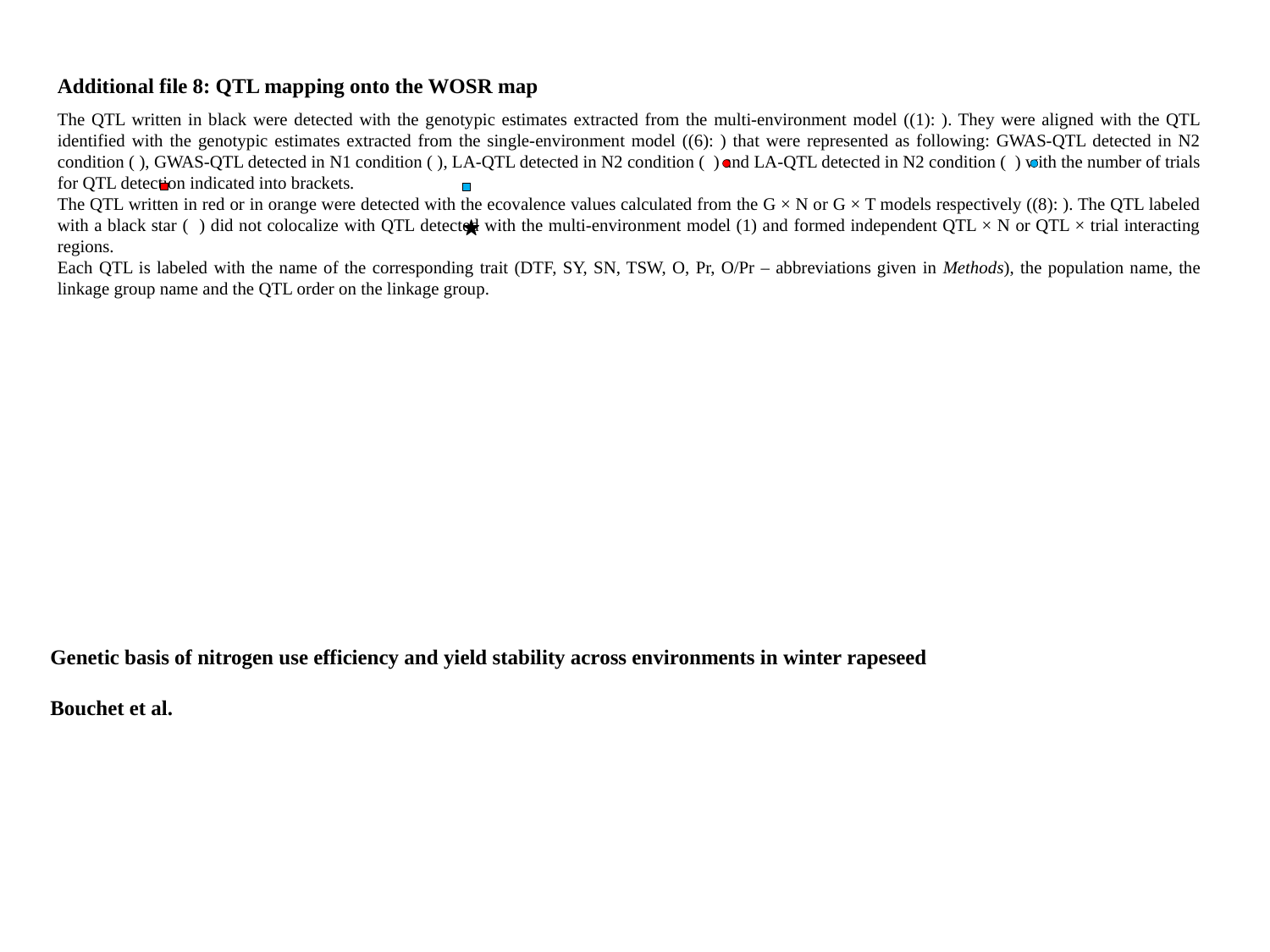

Genetic basis of nitrogen use efficiency and yield stability across environments in winter rapeseed
Bouchet et al.

## Slide 2
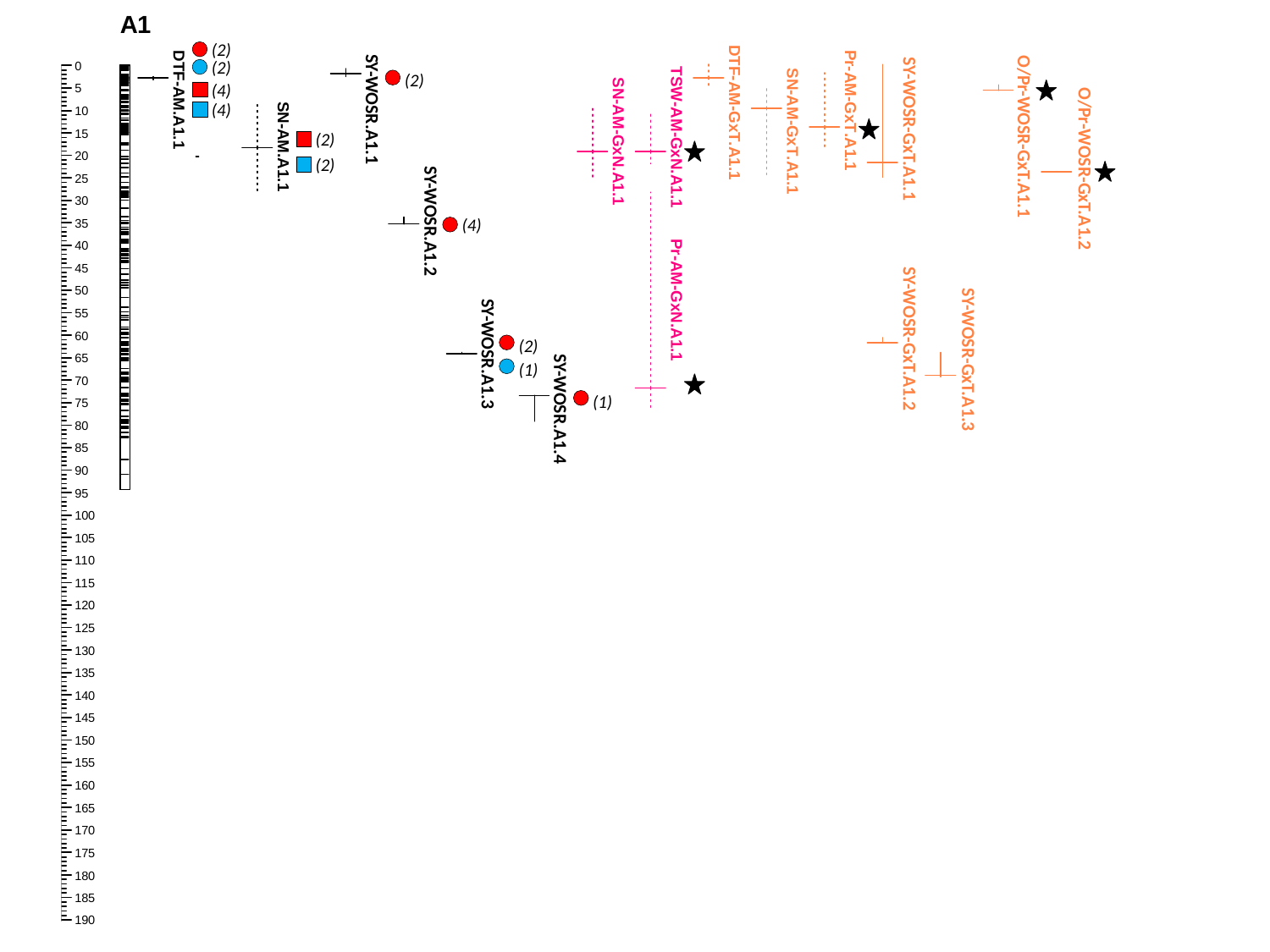

(2)
SY-WOSR.A1.1
O/Pr-WOSR-GxT.A1.1
SY-WOSR-GxT.A1.1
(2)
(2)
O/Pr-WOSR-GxT.A1.2
(4)
(4)
(2)
SY-WOSR.A1.2
(2)
(4)
SY-WOSR-GxT.A1.2
SY-WOSR-GxT.A1.3
SY-WOSR.A1.3
(2)
SY-WOSR.A1.4
(1)
(1)

## Slide 3
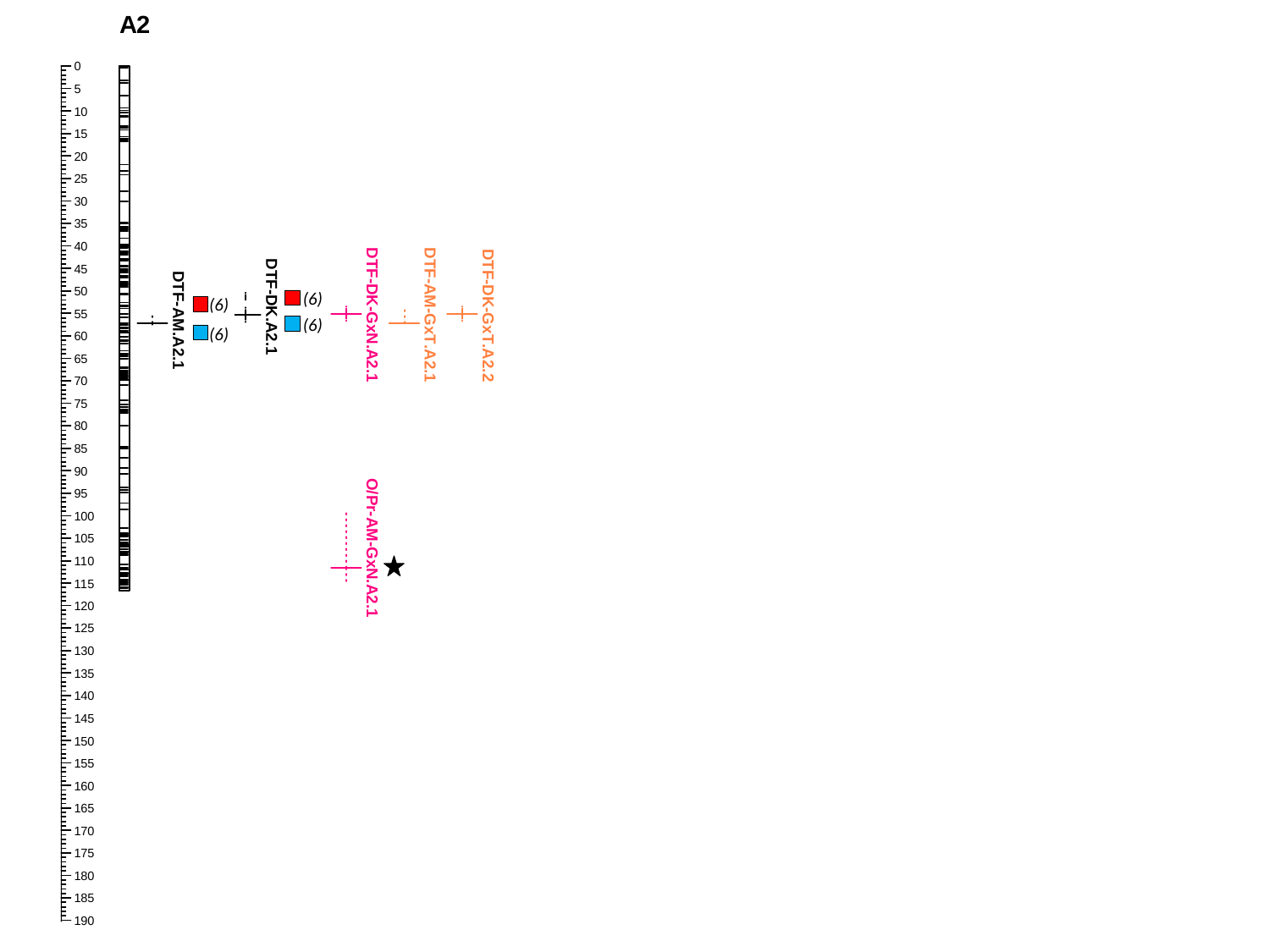

(6)
(6)
(6)
(6)

## Slide 4
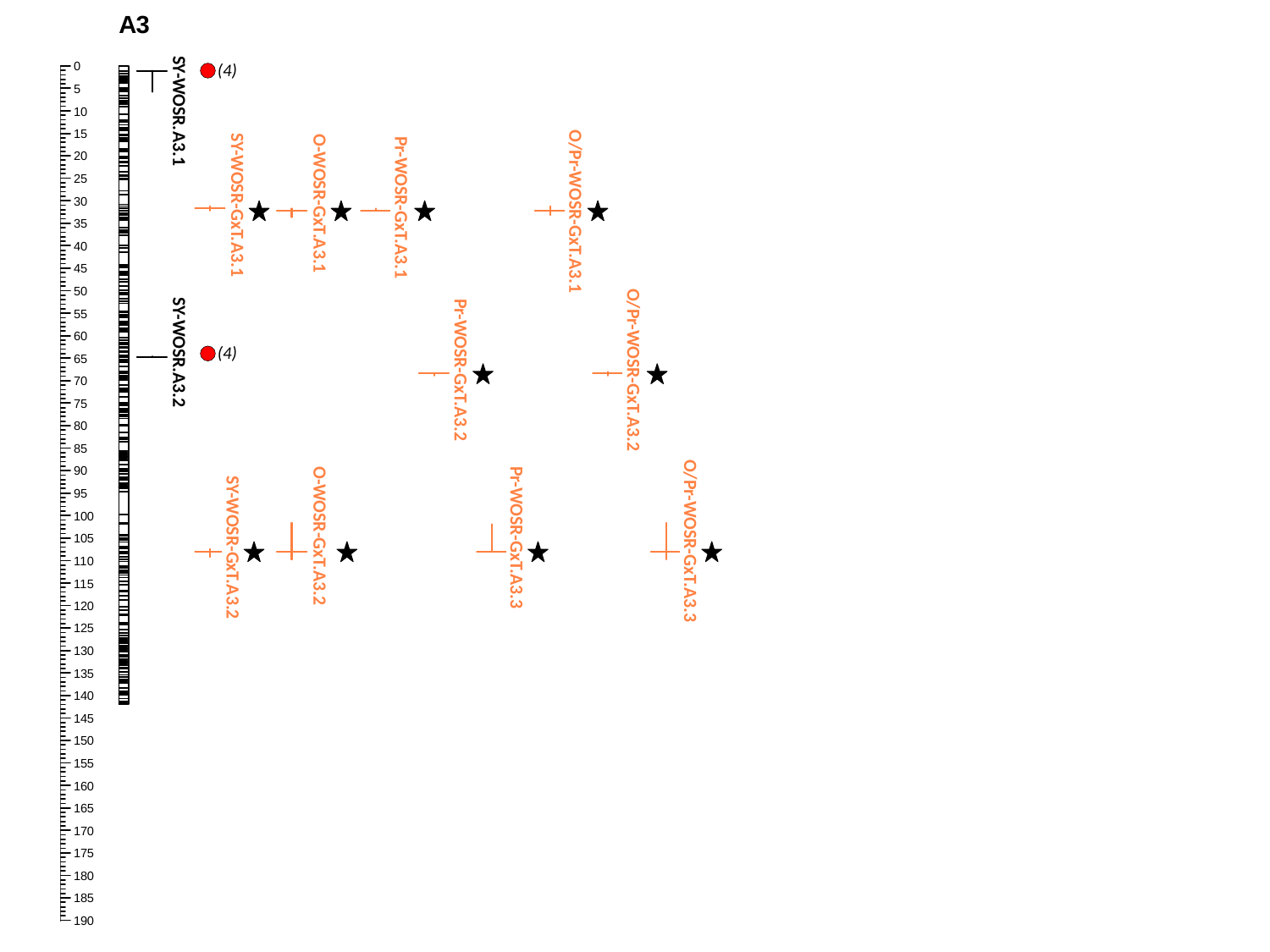

O-WOSR-GxT.A3.1
O-WOSR-GxT.A3.2
O/Pr-WOSR-GxT.A3.1
Pr-WOSR-GxT.A3.1
O/Pr-WOSR-GxT.A3.2
Pr-WOSR-GxT.A3.2
O/Pr-WOSR-GxT.A3.3
Pr-WOSR-GxT.A3.3
SY-WOSR.A3.1
(4)
SY-WOSR-GxT.A3.1
SY-WOSR.A3.2
(4)
SY-WOSR-GxT.A3.2

## Slide 5
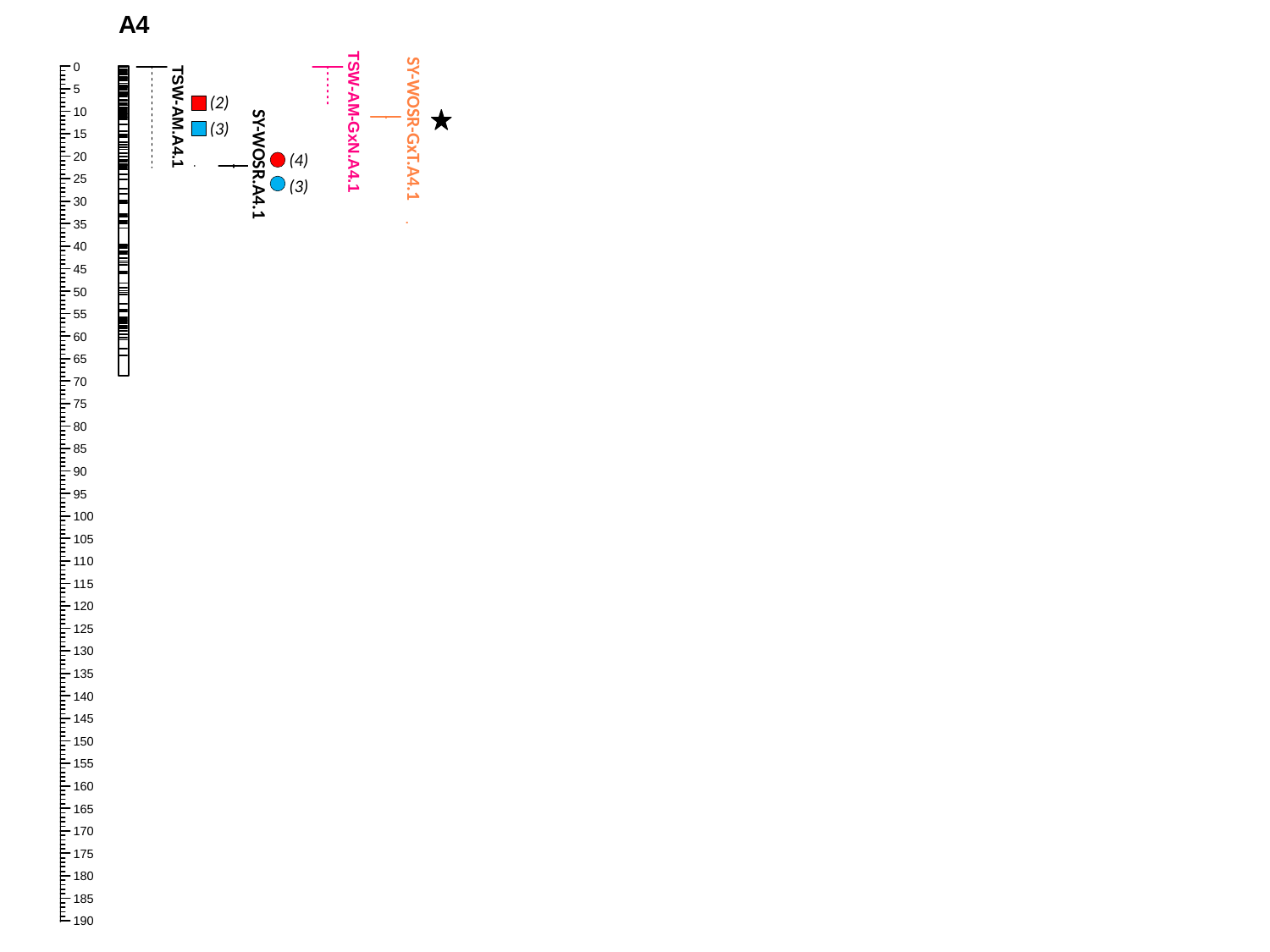

SY-WOSR-GxT.A4.1
(2)
SY-WOSR.A4.1
(3)
(4)
(3)

## Slide 6
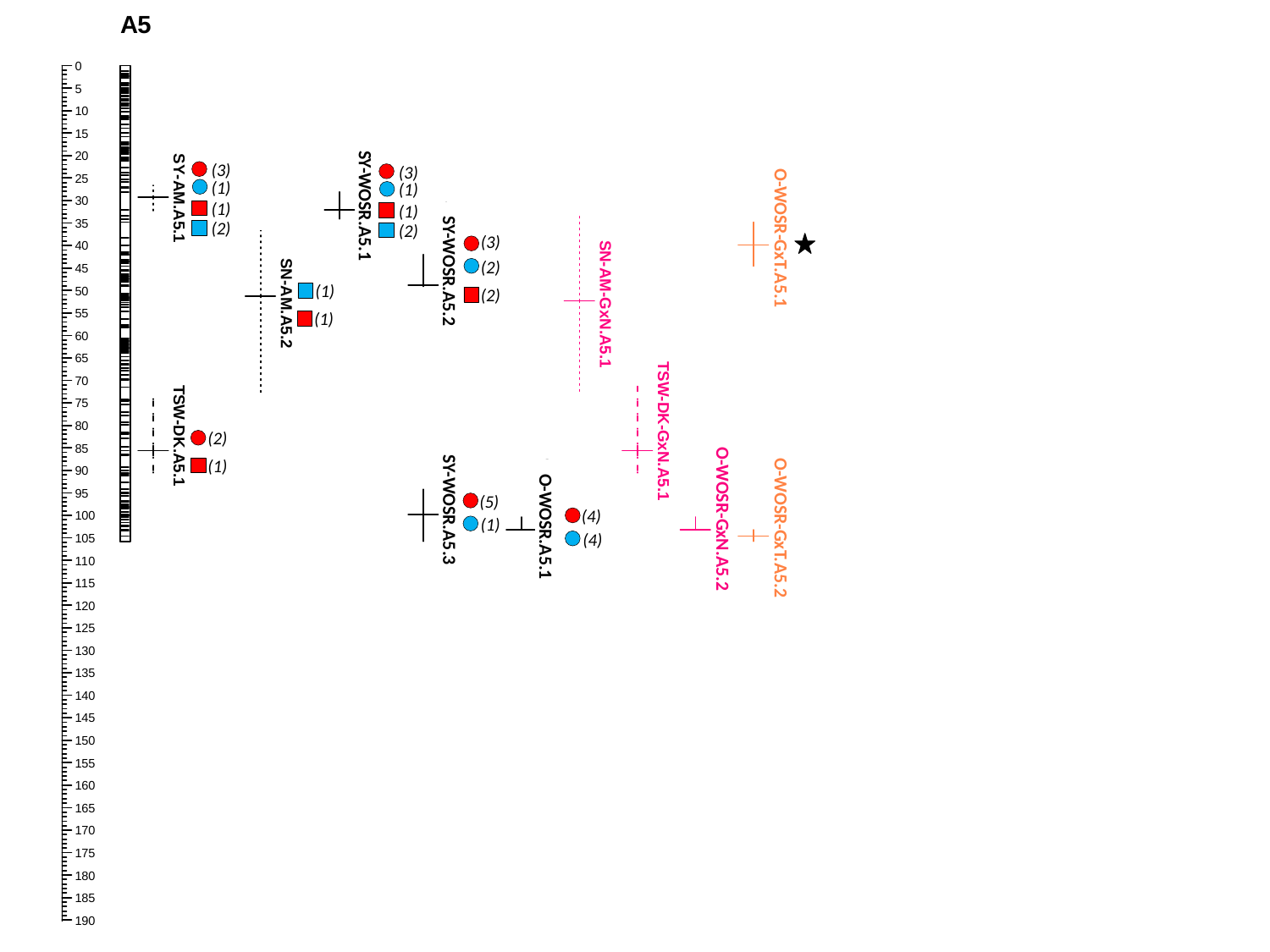

SY-WOSR.A5.1
O-WOSR-GxT.A5.1
(3)
(3)
(1)
(1)
(1)
(1)
SY-WOSR.A5.2
(2)
(2)
(3)
(2)
(1)
(2)
(1)
(2)
O-WOSR-GxN.A5.2
SY-WOSR.A5.3
O-WOSR-GxT.A5.2
(1)
O-WOSR.A5.1
(5)
(4)
(1)
(4)

## Slide 7
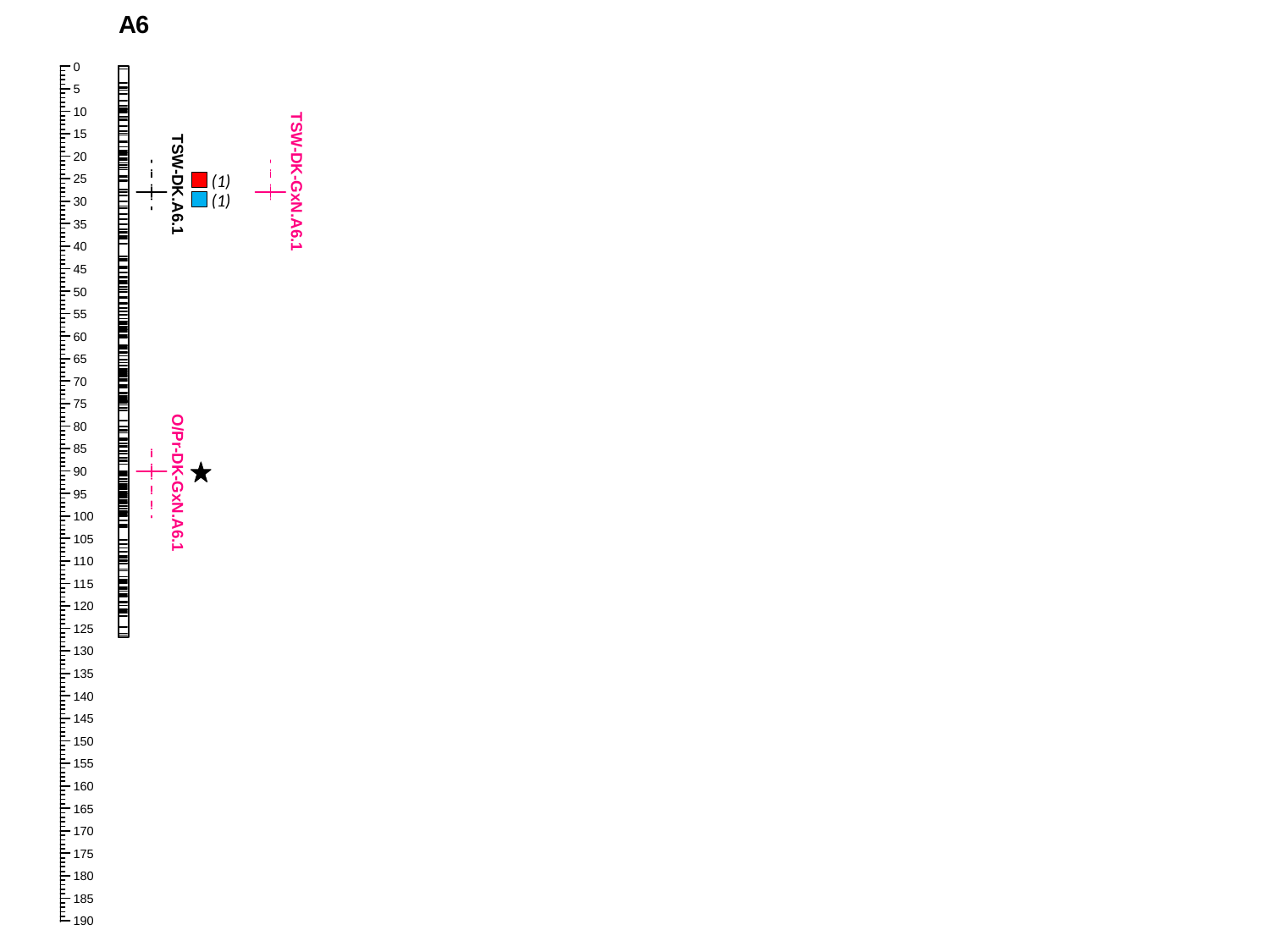

(1)
(1)

## Slide 8
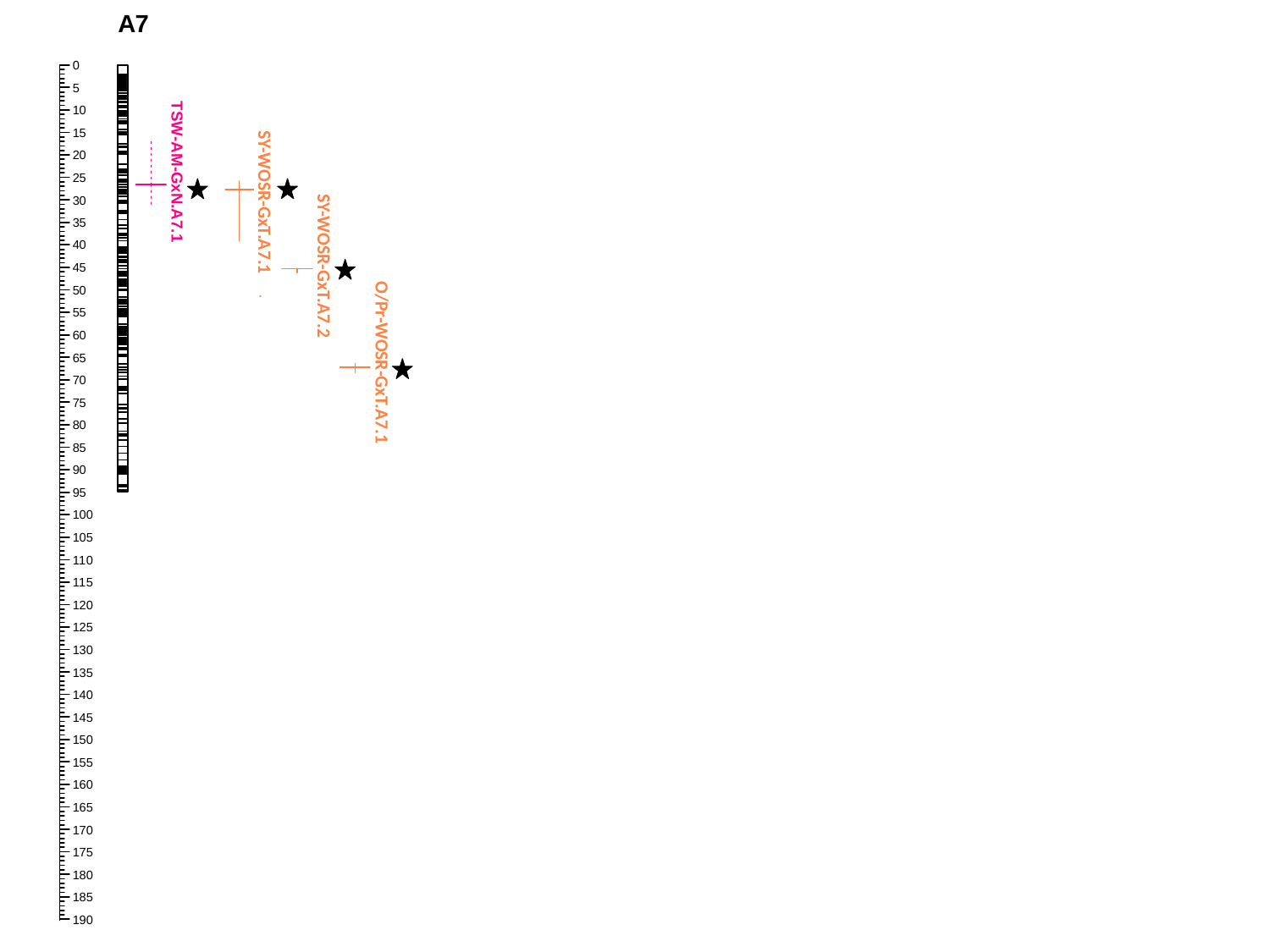

SY-WOSR-GxT.A7.1
SY-WOSR-GxT.A7.2
O/Pr-WOSR-GxT.A7.1

## Slide 9
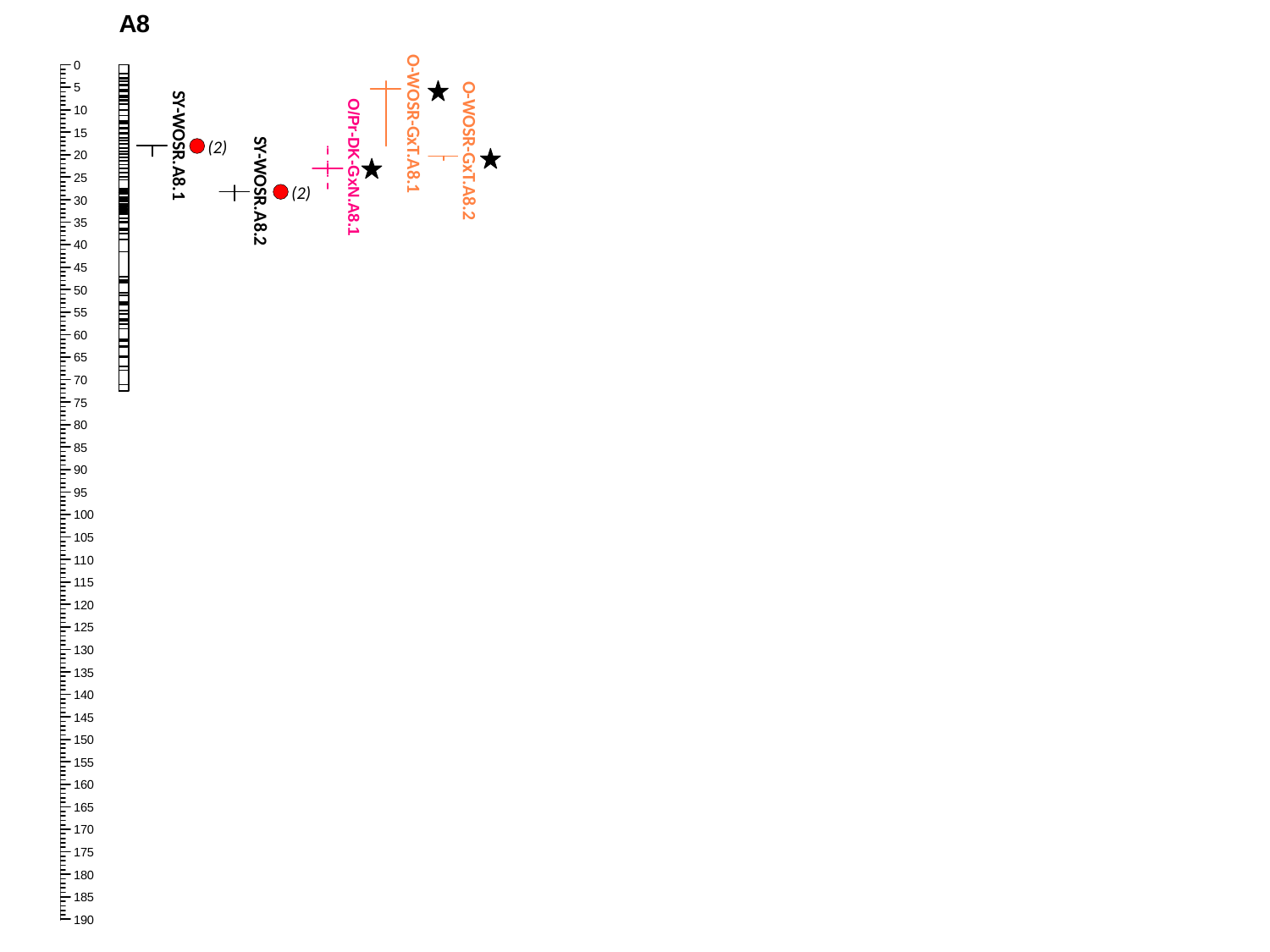

O-WOSR-GxT.A8.1
O-WOSR-GxT.A8.2
SY-WOSR.A8.1
SY-WOSR.A8.2
(2)
(2)

## Slide 10
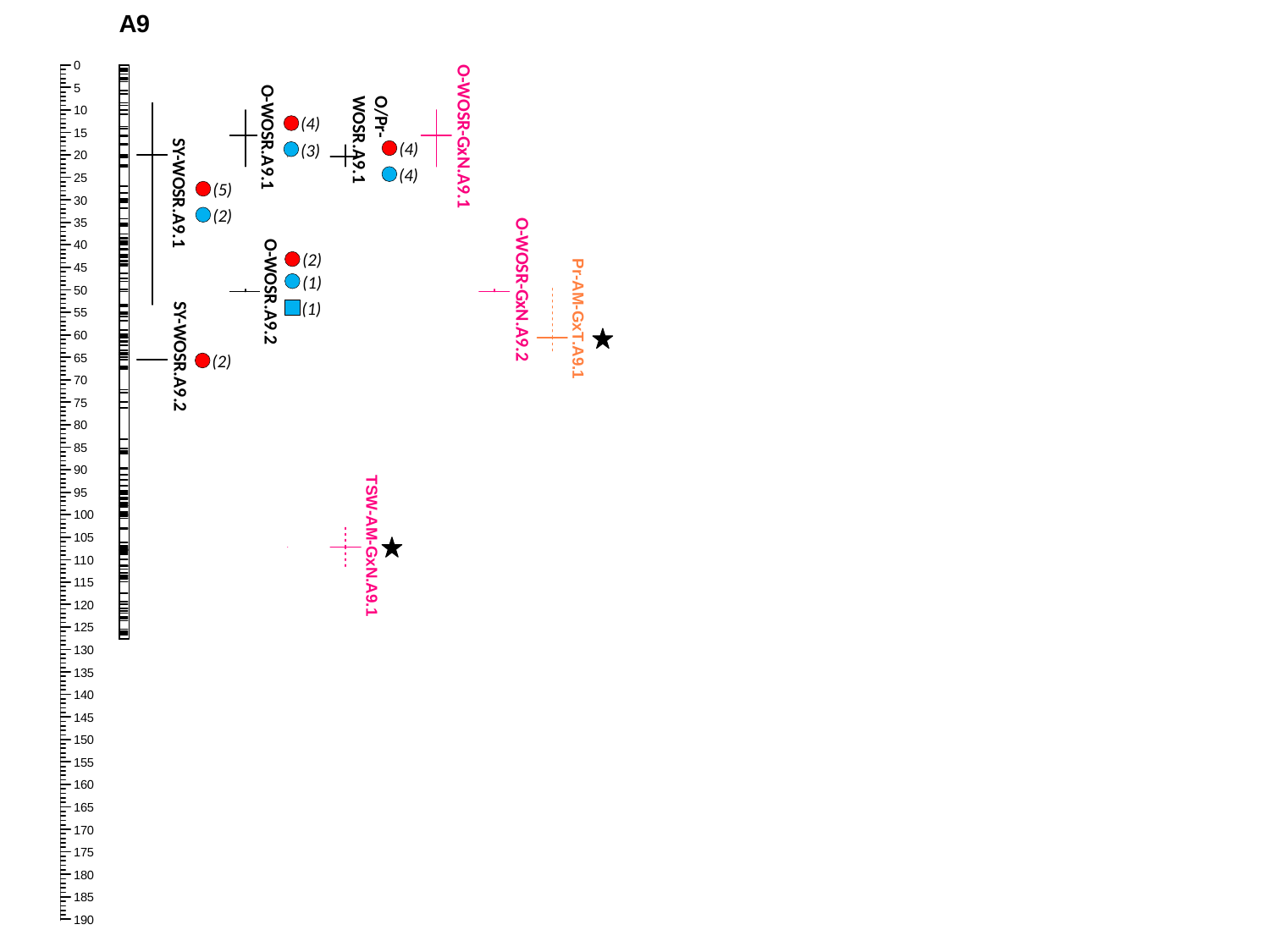

O-WOSR-GxN.A9.1
O-WOSR.A9.1
O/Pr-WOSR.A9.1
(4)
SY-WOSR.A9.1
(4)
(3)
(4)
(5)
(2)
O-WOSR-GxN.A9.2
O-WOSR.A9.2
(2)
(1)
SY-WOSR.A9.2
(1)
(2)

## Slide 11
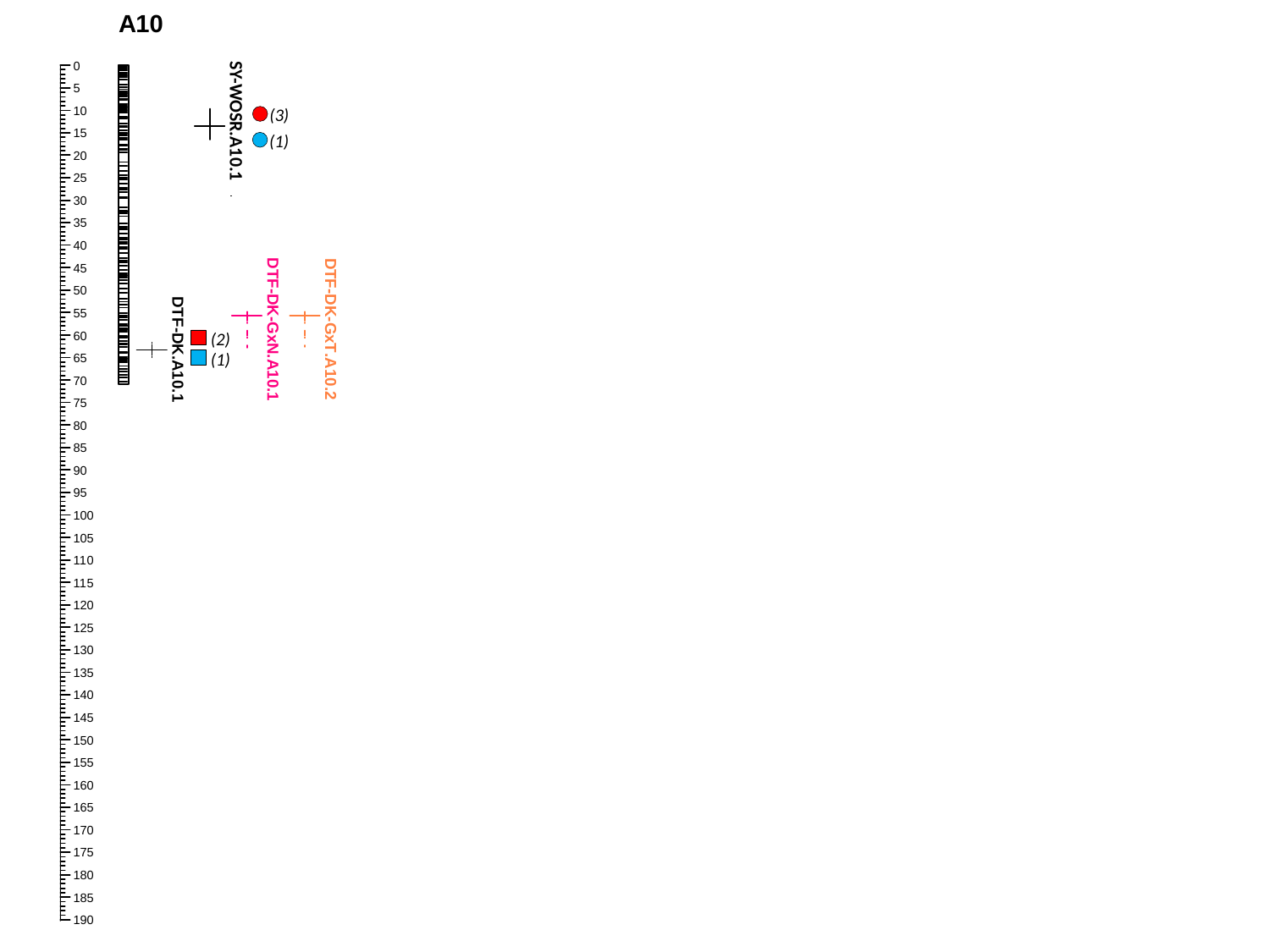

SY-WOSR.A10.1
(3)
(1)
(2)
(1)
(2)
(1)

## Slide 12
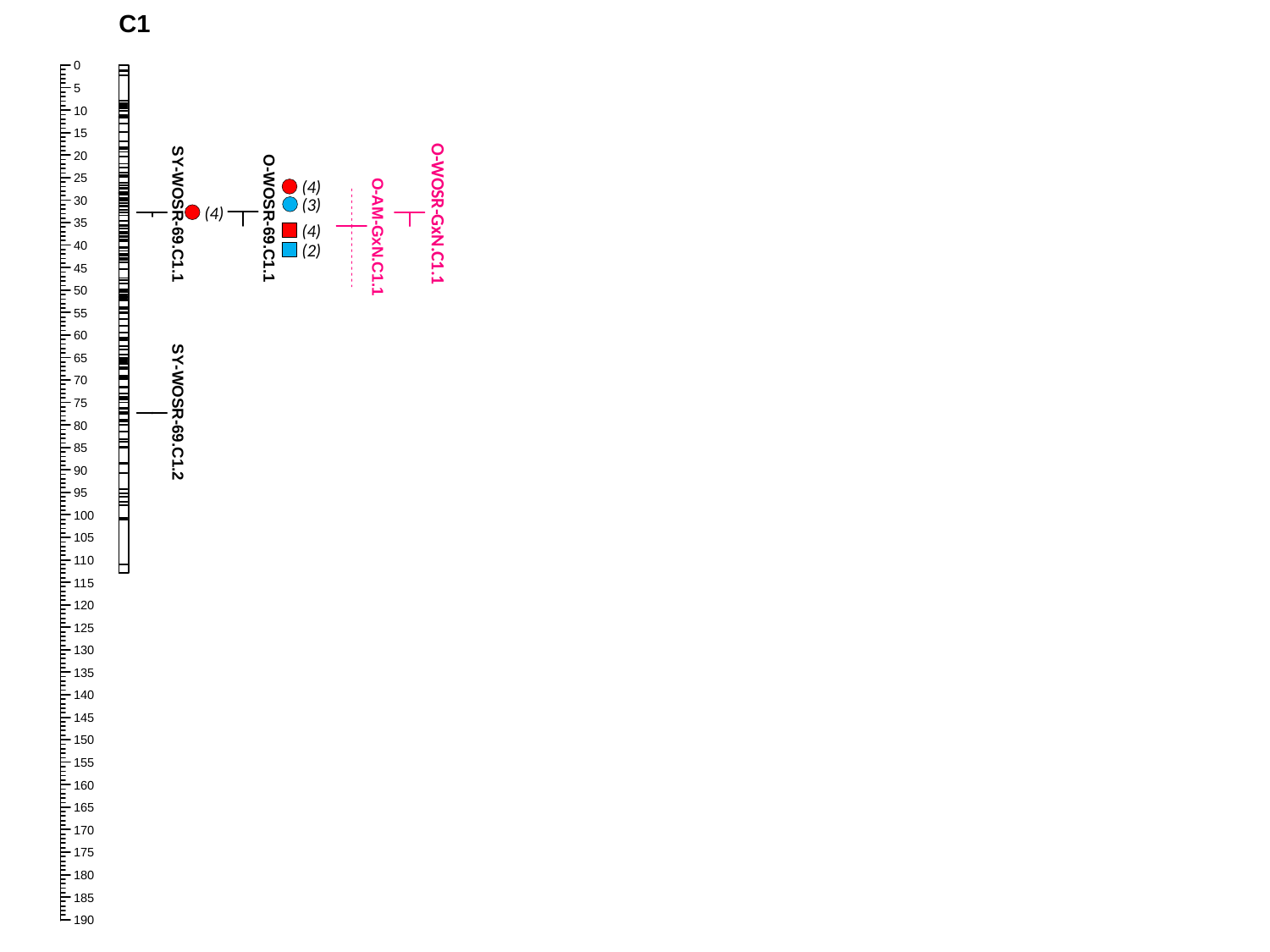

O-WOSR-GxN.C1.1
(4)
(3)
(4)
(4)
(2)

## Slide 13
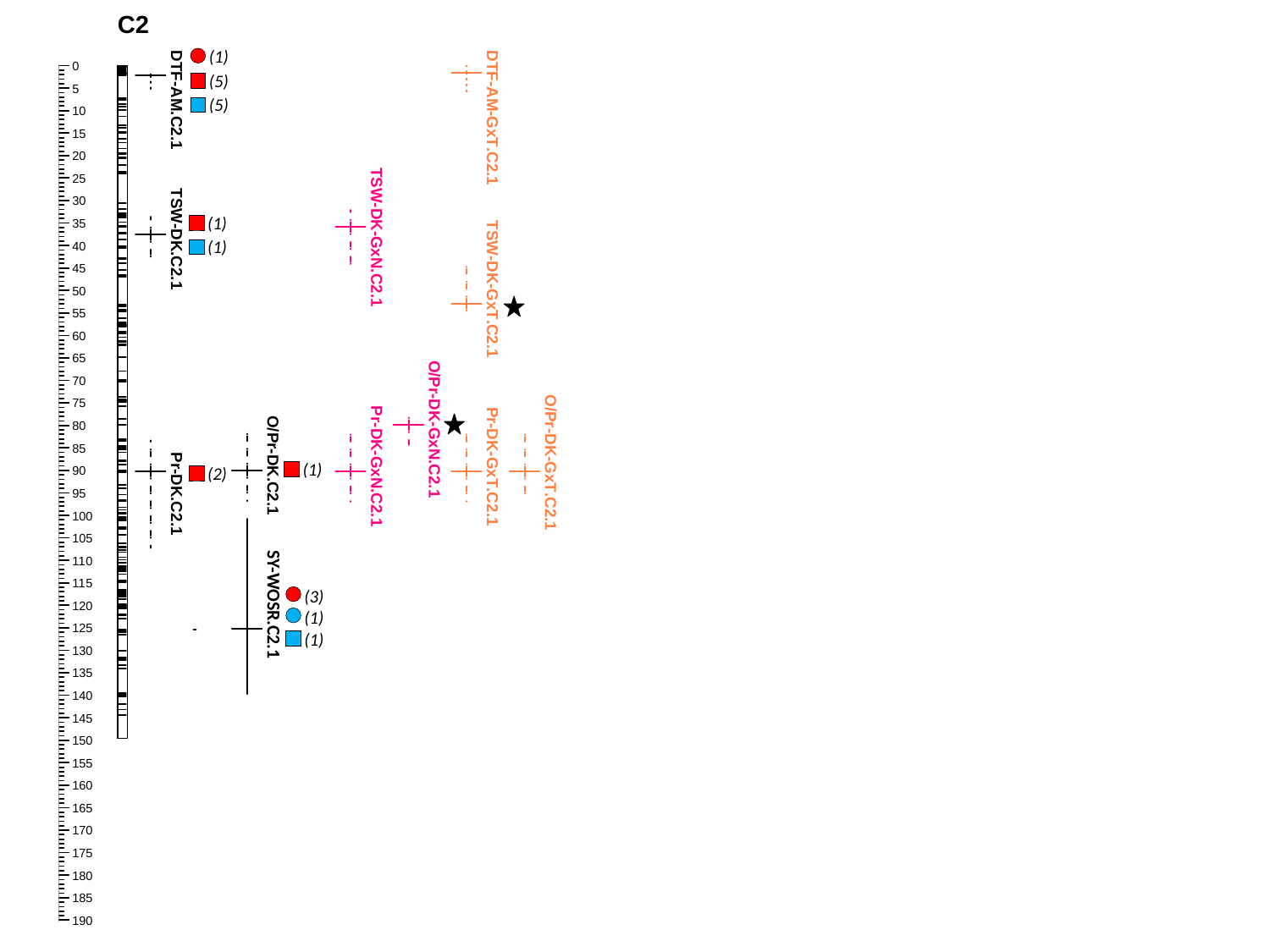

SY-WOSR.C2.1
(3)
(1)
(1)
(1)
(5)
(5)
(1)
(1)
(1)
(2)

## Slide 14
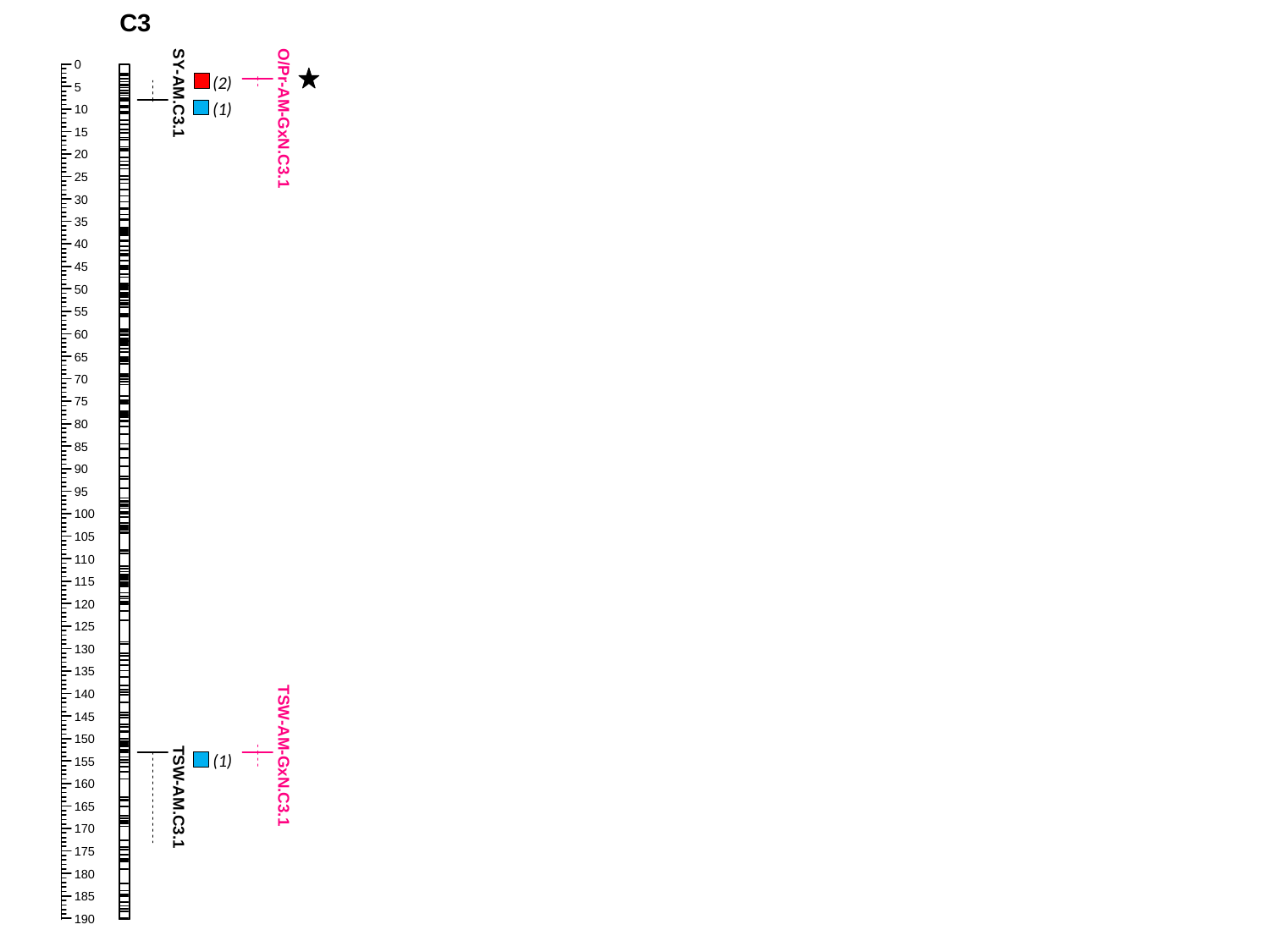

(2)
(1)
(1)

## Slide 15
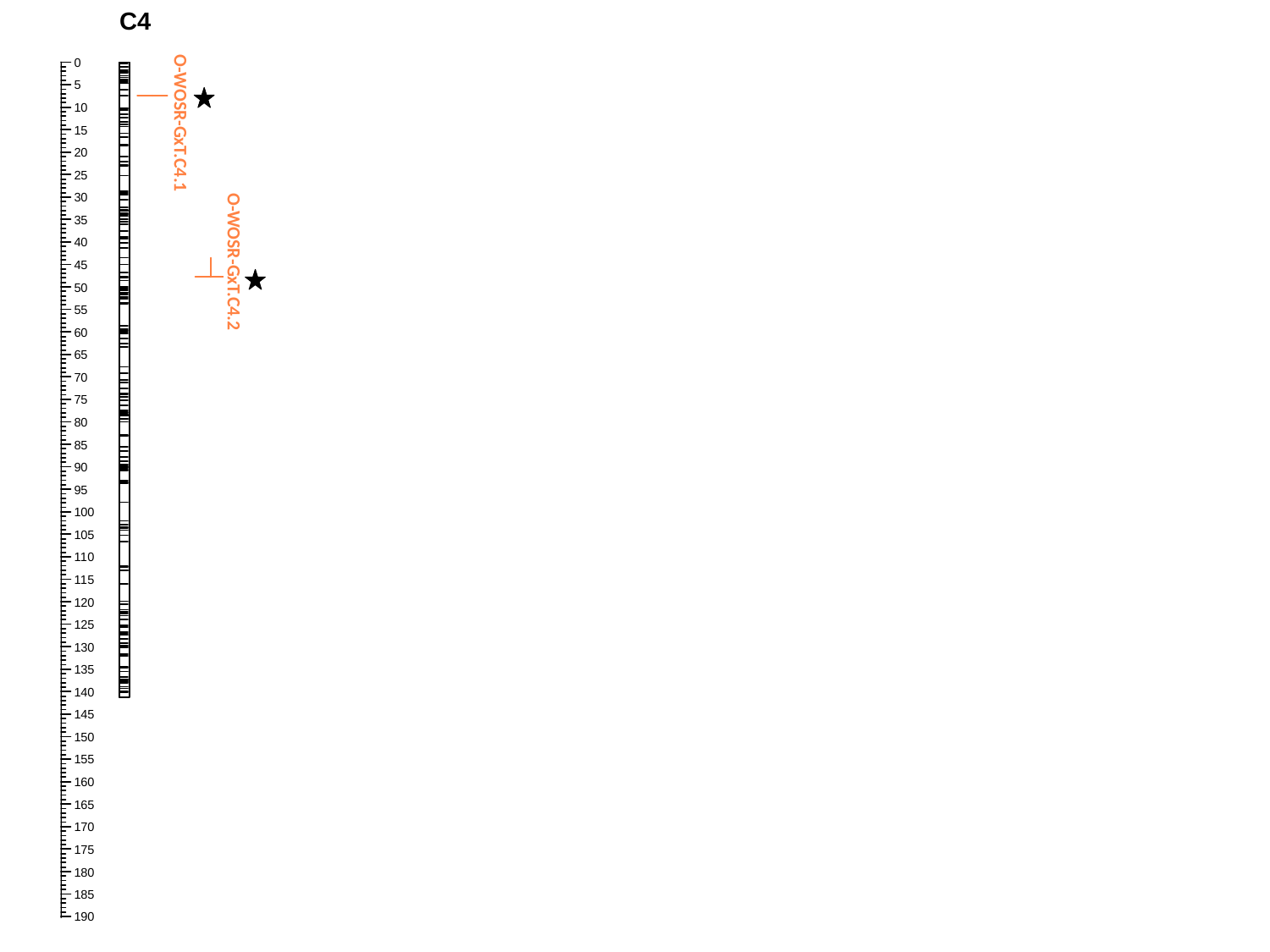

O-WOSR-GxT.C4.1
O-WOSR-GxT.C4.2

## Slide 16
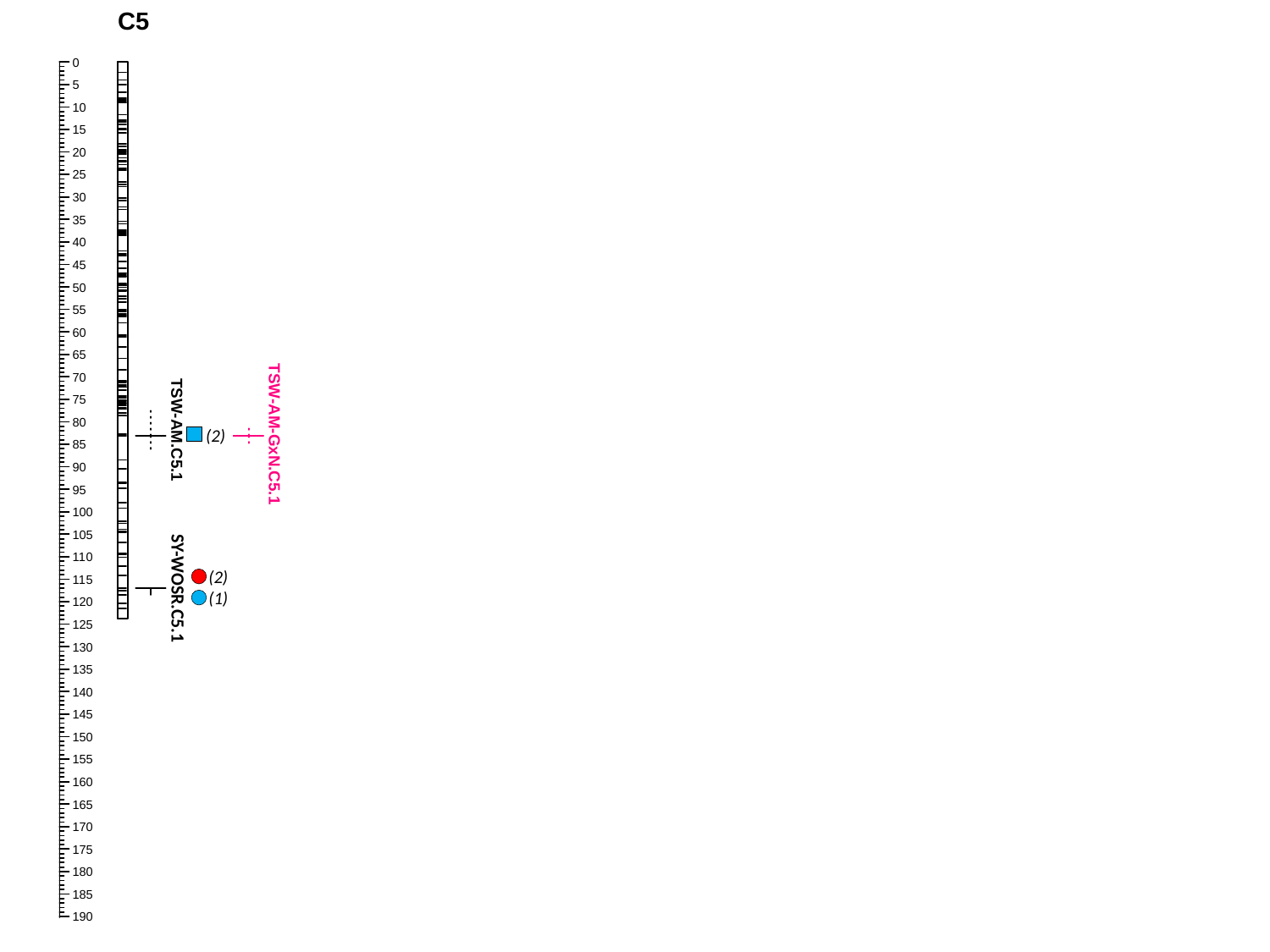

SY-WOSR.C5.1
(2)
(1)
(2)

## Slide 17
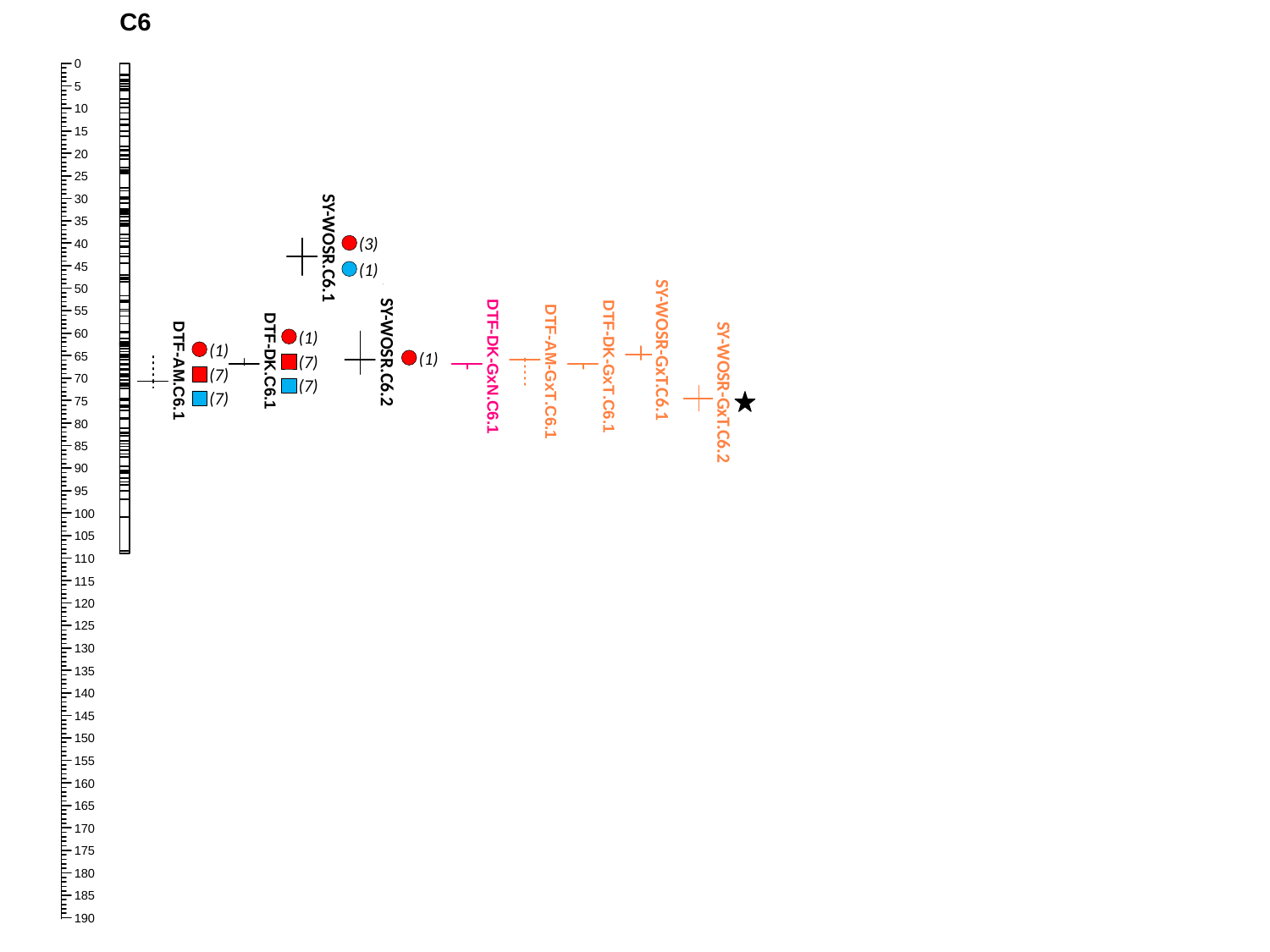

SY-WOSR-GxT.C6.1
SY-WOSR-GxT.C6.2
SY-WOSR.C6.1
(3)
(1)
SY-WOSR.C6.2
(1)
(1)
(1)
(7)
(7)
(7)
(7)

## Slide 18
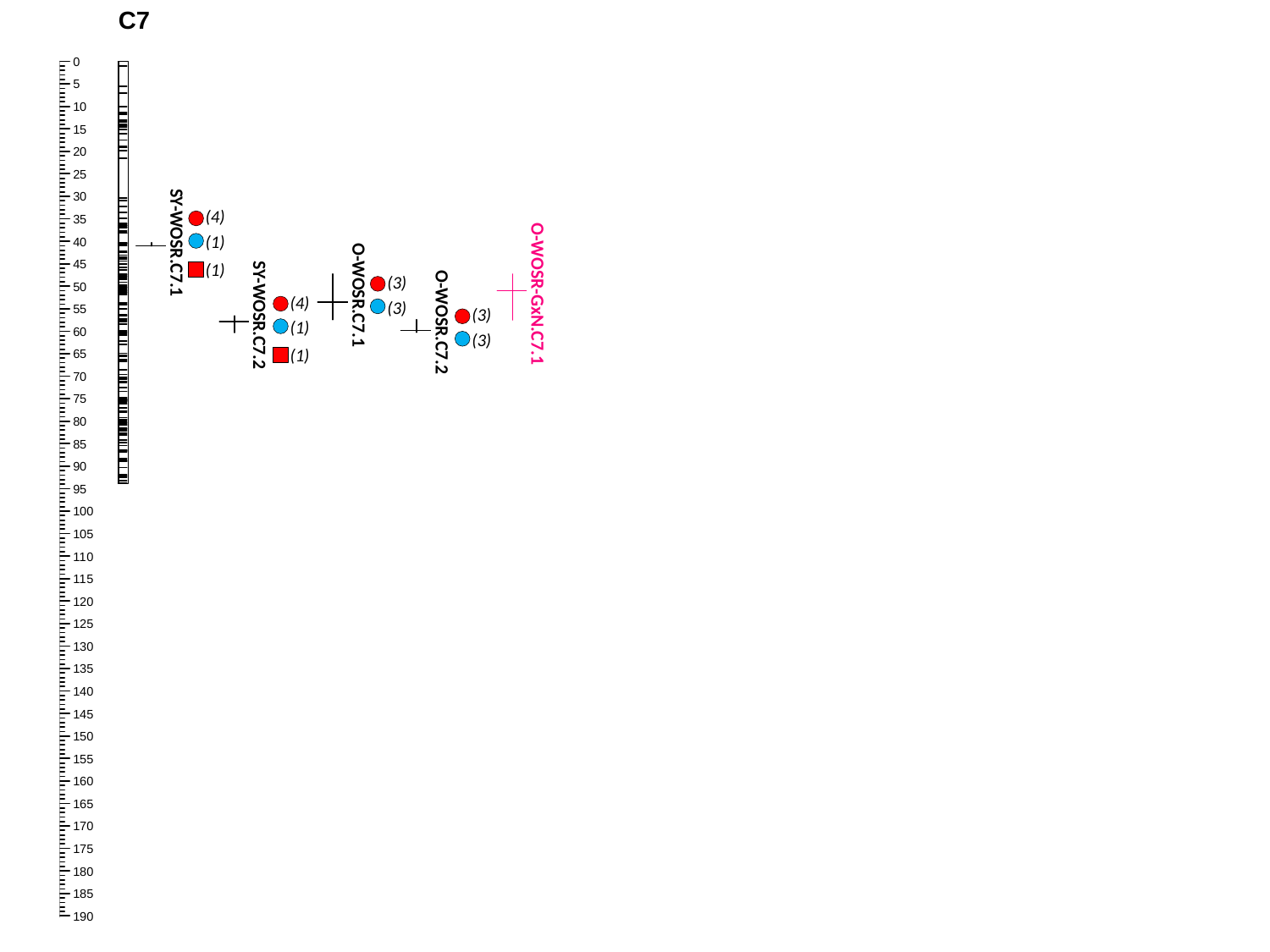

SY-WOSR.C7.1
(4)
O-WOSR-GxN.C7.1
(1)
O-WOSR.C7.1
SY-WOSR.C7.2
O-WOSR.C7.2
(1)
(3)
(4)
(3)
(3)
(1)
(3)
(1)

## Slide 19
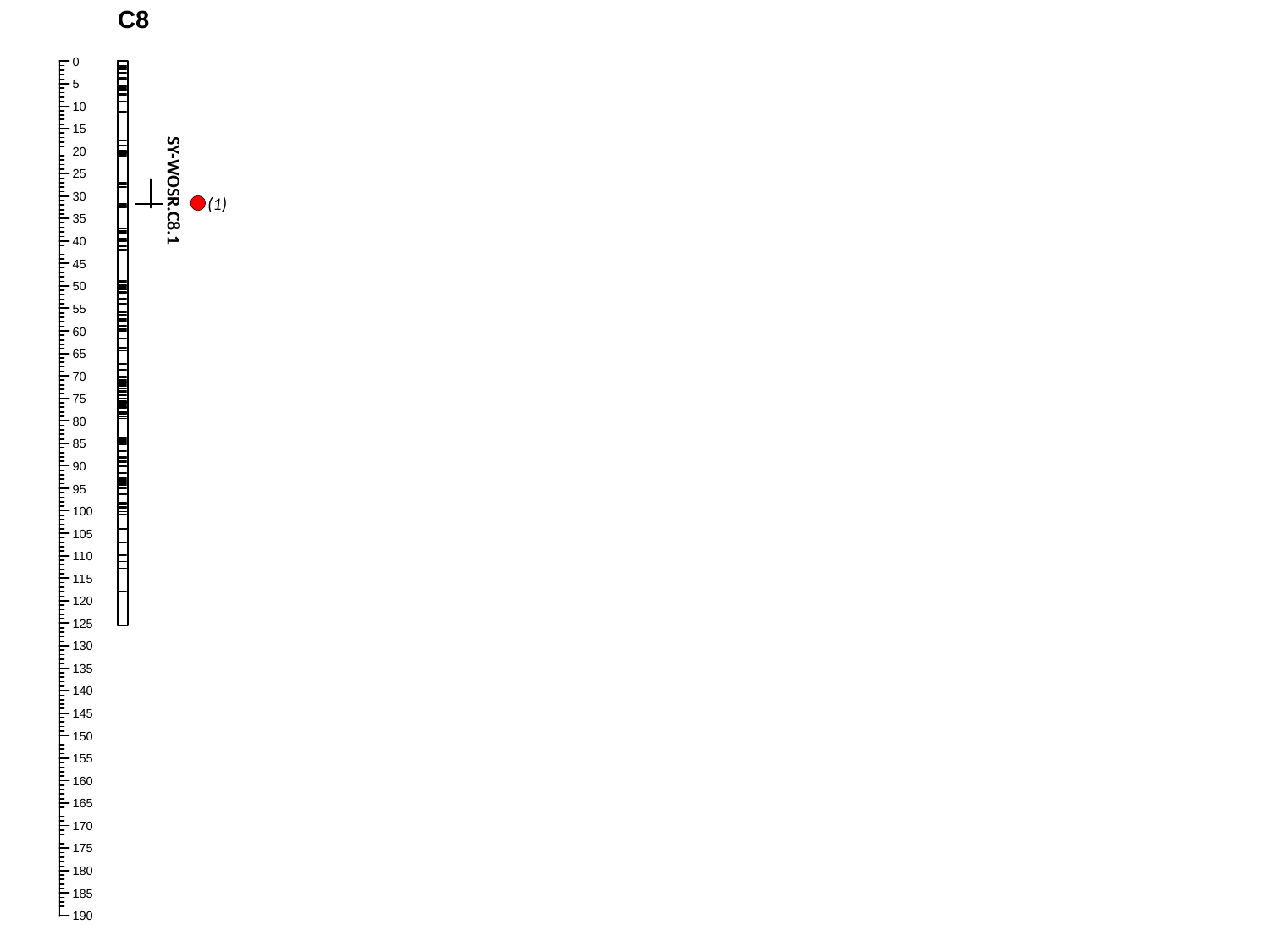

SY-WOSR.C8.1
(1)

## Slide 20
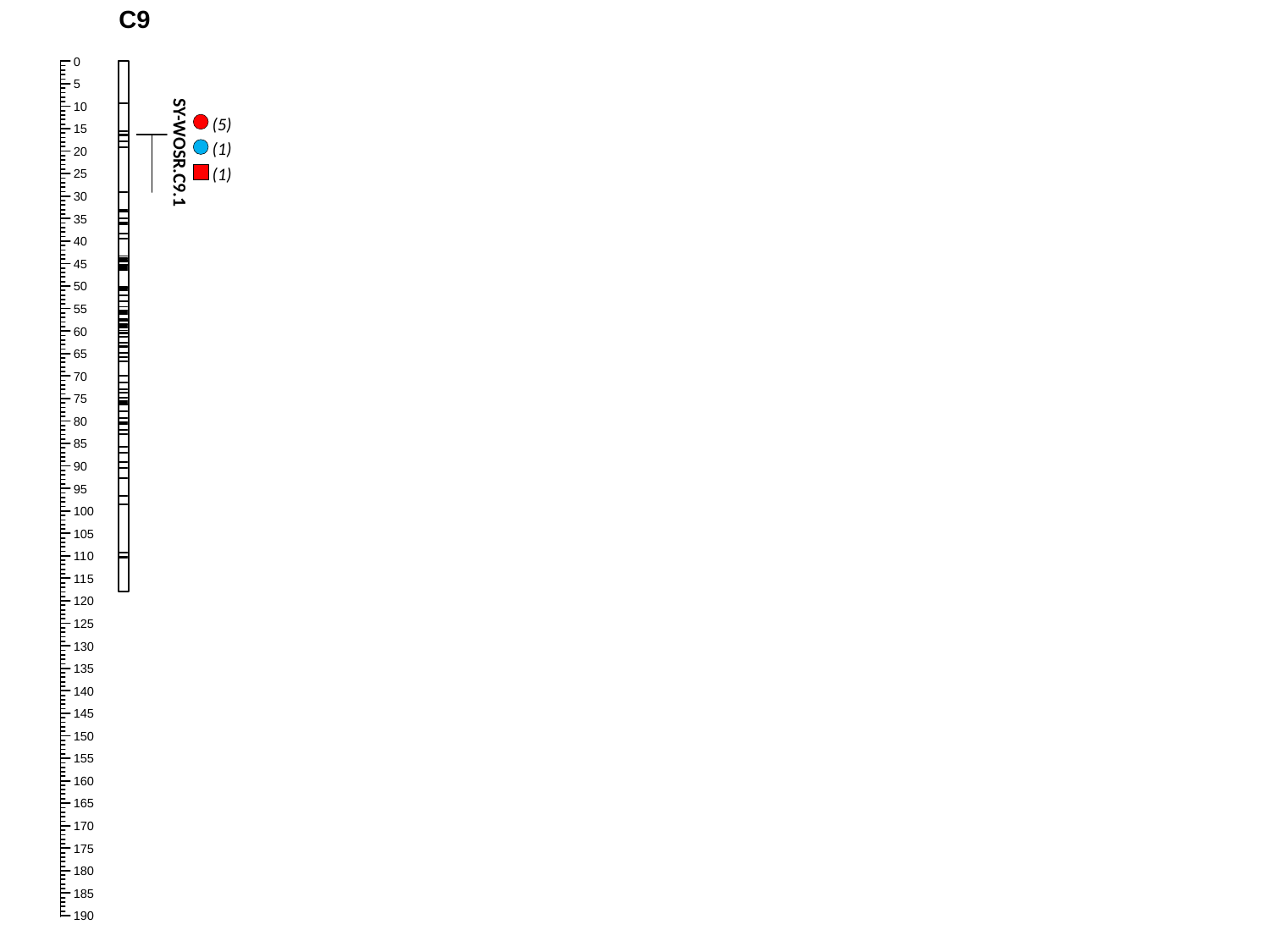

SY-WOSR.C9.1
(5)
(1)
(1)
